# Altered APE1 activity on abasic ribonucleotides is mediated by changes in the nucleoside sugar pucker

**DOI:** 10.1016/j.csbj.2021.05.035

**Published:** 2021-05-25

**Authors:** Nicole M. Hoitsma, Timothy H. Click, Pratul K. Agarwal, Bret D. Freudenthal

**Affiliations:** a Department of Biochemistry and Molecular Biology, University of Kansas Medical Center, Kansas City, KS 66160, USA; b Department of Physiological Sciences and High-Performance Computing Center, Oklahoma State University, Stillwater, OK 74078, USA

**Keywords:** DNA repair, APE1, AP-Endonuclease, Apurinic/apyrimidinic sites, Abasic ribonucleotides, Structural biology

## Abstract

Ribonucleotides (rNTPs) are predicted to be incorporated into the genome at a rate of up to 3 million times per cell division, making rNTPs the most common non-standard nucleotide in the human genome. Typically, misinserted ribonucleotides are repaired by the ribonucleotide excision repair (RER) pathway, which is initiated by RNase H2 cleavage. However, rNTPs are susceptible to spontaneous depurination generating abasic ribonucleotides (rAPs), which are unable to be processed by RNase H2. Additionally, rAPs have been found in nascent RNA and coupled to R-loops. Recent work identified that base excision repair (BER) protein AP-Endonuclease 1 (APE1) is responsible for the initial processing of rAPs embedded in DNA and in R-loops. APE1 is a well characterized AP endonuclease that cleaves 5′ of abasic sites, but its ability to cleave at rAPs remains poorly understood. Here, we utilize enzyme kinetics, X-ray crystallography, and molecular dynamics simulations to provide insight into rAP processing by APE1. Enzyme kinetics were used to determine pre-steady-state rates of APE1 cleavage on DNA substrates containing rAP, revealing a decrease in activity compared to cleavage at a canonical deoxy-AP substrate. Using X-ray crystallography, we identified novel contacts between the rAP and the APE1 active site. We demonstrate that the rAP sugar pucker is accommodated in the active site in a C3′-endo conformation, influencing its position and contributing to a decrease in activity compared to the deoxy-AP site. Together, this work provides molecular level insights into rAP processing by APE1 and advances our understanding of ribonucleotide processing within genomic DNA.

## Graphical abstract

**Figure:**
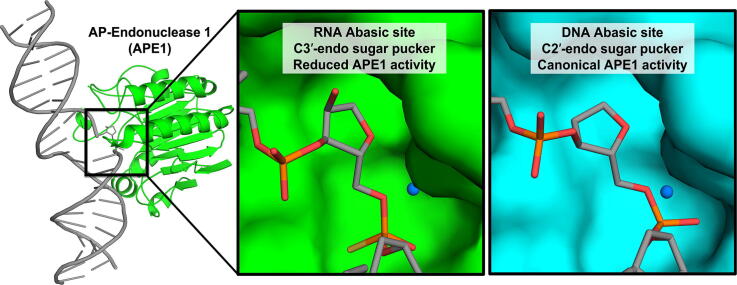

## 1 Introduction

To maintain genomic integrity the cellular DNA must be faithfully replicated during each round of cell division. DNA replication is carried out by DNA polymerases, which use deoxynucleoside triphosphates (dNTPs) as substrates to extend the DNA strand one nucleotide at a time. However, due to the high abundance of ribonucleoside triphosphates (rNTPs) in the cellular nucleotide pool, DNA polymerases frequently misincorporate rNTPs into the genome [1]. Based on the ribonucleotide incorporation propensities of human replicative polymerases, Pol ε and Pol δ, it is predicted that ribonucleotides may be incorporated into the genome at a rate of up to three million ribonucleotides per cell division [2], [3], [4]. The frequent incorporation of ribonucleotides in DNA makes them the most common type of non-standard or ‘damaged’ nucleotide found within the genome. Ribonucleotides embedded in the genome are repaired by the ribonucleotide excision repair pathway (RER). This repair pathway is initiated by the RNase H2 enzyme, which cleaves 5′ of the ribose sugar backbone [5]. However, recent work identified that damaged ribonucleotides, such as abasic ribonucleotides (rAP), cannot be processed by RNase H2 [6]. Instead, rAPs are processed by AP-Endonuclease 1 (APE1), a multifunctional enzyme in the base excision repair (BER) pathway [6]. During BER, abasic (AP) sites are first processed by APE1, which cleaves 5′ of the abasic site generating 3′ hydroxyl and 5′ deoxyribose phosphate termini. Following cleavage by APE1, the downstream BER proteins, DNA polymerase β and XRCC1/Ligase IV, process the 5′ nick, ultimately restoring the coding potential of the DNA [7], [8], [9], [10].

Furthermore, recent work has identified rAP sites present in a number of diverse and biologically relevant nucleic acid structures, such as nascent, messenger, and ribosomal RNA with a prevalence of approximately three RNA abasic sites per million ribonucleotides [11]. RNA abasic sites were found to be coupled to R-loops, three stranded nucleic acid structures composed of a DNA:RNA hybrid and a single-stranded DNA. These structures often occur when nascent mRNA re-hybridizes to its template DNA strand, thus displacing the non-complementary strand of single-stranded DNA [12], [13]. In this way, R-loops form naturally during transcription and act as regulators of gene expression, but their unplanned or persistent formation can be a risk to genome stability [14]. Once formed, R-loops are particularly stable, as RNA/DNA interactions are thermodynamically more stable than DNA/DNA interactions [15]. Additionally, the 2′ OH group of ribose confers stability to abasic sites in RNA (ribo-AP) compared to deoxyribose in DNA (deoxy-AP) [16]. Data suggests that R-loops facilitate the formation of RNA abasic sites which, given the stability of R-loops and RNA abasic sites, may affect how long a particular R-loop persists, and thus influence the expression of the transcript and its downstream substrates [11], [17], [18], [19]. To repair RNA abasic sites in R-loops, APE1 is recruited to cleave the RNA abasic site which facilitates resolution of the R-loop [11]. If these R-loops are not resolved they can block transcription of the DNA and promote replication fork collapse [12], [20].

Despite the importance of understanding how abasic ribonucleotides are processed, the molecular basis for rAP site recognition and cleavage by APE1 remains unknown. Here, we study how rAP sites are processed by APE1 using a combination of kinetic, structural, and computational approaches. Our results highlight the effect of the ribonucleotide 2 OH in altering APE1 cleavage activity. We find that the sugar pucker of the rAP substrate influences its position in the active site and contributes to a decrease in cleavage activity compared to the deoxy-AP site. Together, these results further our understanding of how damaged ribonucleotides are processed and repaired in the genome.

## 2 Materials and methods

### 2.1 DNA sequences

All oligonucleotide sequences were purchased from and purified by IDT, before being resuspended and annealed. Annealing reactions were performed by mixing the oligonucleotides in a 1:1 ratio, heating the mixture to 95 °C for 5 minutes, and then allowing the reaction to cool at a rate of 1 °C min^−1^ to 4 °C. To generate the 21-mer DNA for crystallization of the APE1 product complex, the following DNA sequences were used: nondamaged strand, 5′-GGA-TCC-GTC-GAG-CGC-ATC-AGC-3′, damaged strand, 5′-GCT-GAT-GCG-CXC-GAC-GGA-TCC-3′, where the underlined X represents a rAP analog, tetrahydrofuran with an additional 2′ OH. For substrate complex crystallization a phosphorothioate modification (indicated by /) was included to prevent cleavage. Substrate complex crystallization utilized a 21-mer with the following sequences: nondamaged strand, 5′-GGA-TCC-GTC-GAG-CGC-ATC-AGC-3′; damaged strand, 5′-GCT-GAT-GCG-C/XC-GAC-GGA-TCC-3′. To generate the 30-mer used for kinetic and binding experiments, the following sequences were used: nondamaged strand, 5′-ATG-CGG-ATC-CGT-CGA-GCG-CAT-CAG-CGA-ACG-3′, damaged strand with a 5′ fluorescein label (indicated by asterisk), 5′-*CGT-TCG-CTG-ATG-CGC-XCG-ACG-GAT-CCG-CAT-3′.

### 2.2 Protein expression and purification

Human wild type and truncated APE1 C138A were expressed and purified as previously described [21]. Briefly, using a pET system, the APE1 gene was overexpressed in BL21-CodonPlus (DE3)-RP *Escherichia coli* and cells were lysed by sonication. APE1 protein was purified from the cell lysate using heparin, cation exchange, and gel filtration resins on an ATKA-Pure FPLC. Purity was confirmed by SDS-PAGE analysis and concentration determined by absorbance at 280 nm.

### 2.3 Crystallization and structure determination

APE1 protein used for crystallography contains a truncation of the N-terminal 42 amino acids and the C138A mutation to aid in crystallization (referred to as ΔAPE1) [22]. ΔAPE1 is regularly used for structural determination due to the unstructured nature of the N-terminal region which prevents its crystallization [23]. Here, we are focused on structural effects in the active site (APE1 has been shown to use the same active site for processing rAP, as its canonical deoxy-endonuclease function [6]), which does not include portions of the N-terminal domain. For structural studies, annealed DNA was mixed with ΔAPE1 to a final concentration of 0.56 mM DNA and 10–12 mg ml^−1^ (0.28–0.34 mM) ΔAPE1 protein, and then incubated for 30 min at room temperature. The ΔAPE1:DNA complex crystals were generated via vapor diffusion. The reservoir solution for product complex crystal formation was 200 mM MgCl_2_, 100 mM Sodium Citrate (pH 5), and 10–15% PEG 20,000. The reservoir solution for substrate complex crystal formation was 50 mM tri-Sodium Citrate (pH 4), 100 mM NaCl, and 10% PEG 6,000. For X-ray diffraction, ΔAPE1:DNA crystals were transferred to a cryosolution containing reservoir solution with the addition of 25% ethylene glycol. The MnCl_2_ soaks were performed in the same manner with the addition of 50 mM MnCl_2_ to the cryosolution. The crystals were flash frozen and data was collected at 100 K on a Rigaku MicroMax-007 HF rotating anode diffractometer equipped with a Dectris Pilatus3R 200 K-A detector system at a wavelength of 1.54 Å. This allowed for anomalous data detection after phasing by molecular replacement with high redundancy. Data were processed and scaled with the HKL3000R software package. Initial models were determined by molecular replacement with a previously determined ΔAPE1:DNA complex (PDB 5DFF) as a reference. Refinement was carried out with PHENIX, model building with Coot, and figures were prepared with PyMOL (Schrödinger LLC) [24], [25].

### 2.4 Pre-steady-state kinetics

Activity measurements were carried out using a rapid quench-flow (Kintek RQF-3). This equipment allows for APE1 protein and substrate DNA to be mixed and rapidly quenched at specific time intervals. As the APE1 N-terminal domain has been referenced in APE1 activity on RNA, we used full length wild type APE1 in our kinetics studies [26]. The fluorescein-containing rAP DNA substrates were used to measure APE1 incision activity at 37° C. Reactions were carried out in reaction buffer containing 50 mM HEPES (pH 7.5), 100 mM KCl, 5 mM MgCl_2_, and 0.1 mg/ml BSA, and quenched at a range of timepoints between 0.1 and 10 seconds with 200 mM EDTA. In multiple turnover experiments, the final concentration of APE1 protein and DNA was 30 nM and 100 nM, respectively, to allow for multiple cleavage reactions. For the single turnover reactions, the DNA was limiting (50 nM) to APE1 protein (500 nM). The quenched reactions were then diluted 1:1 with loading dye (100 mM EDTA, 80% deionized formamide, 0.25 mg/ml bromophenol blue, and 0.25 mg/ml xylene cyanol), incubated at 95° for 6 min, and then run on a 22% denaturing polyacrylamide gel to separate substrate and product DNA. A Typhoon imager was then used to image the gel, followed by quantification with ImageJ and curve fitting with KaleidaGraph. The biphasic curves (multiple turnover) were fit with the equation: product = A(1 − e^−k^_obs_^t^) + v_ss_t, where A is the amplitude of the rising exponential (represents the fraction of actively bound enzyme) and k_obs_ is the first order rate constant. The steady-state rate constant (k_ss_) is the steady-state velocity (v_ss_)/A. Single turnover data was fit to equation: product = A(1 − e^−k^_obs_^t^). Each time point in the curves represents an average of at least three independent experiments ± the error determined using standard deviation of the mean.

### 2.5 Electrophoretic mobility shift assay (EMSA)

This assay probed the binding interaction of APE1 and a DNA substrate that contained a centrally located rAP site analog (oligonucleotides listed above). Samples were prepared by mixing 2 nM DNA with a varying amount of full length wild-type APE1 (0–20 nM) in buffer containing 50 mM Tris (pH 8), 1 mM EDTA (pH 8), 0.2 mg/ml BSA, and 1 mM DTT. Additionally, 5% v/v sucrose was added to each sample for purposes of gel loading. Samples were equilibrated on ice for 30 min, loaded into a 10% native 59:1 acrylamide:bisacrylamide gel. Native gels were run for 90 min on ice at a constant voltage of 120 V, and then imaged with a Typhoon imager in fluorescence mode. DNA bands were quantified using ImageJ, and curve fitting was done in Kaleidagraph using Eq. (1):

$$AB=\frac{\left(A_{T}+B_{T}+{\text{K}}_{D,app}\right)-\sqrt{{\left(A_{T}+B_{T}\;\text{+}\;{\text{K}}_{D,app}\right)}^{2}-4\left(A_{T}B_{T}\right)}}{2}$$

where A_T_ and B_T_ represent the total concentration of APE1 and DNA, respectively, and AB is the concentration of APE1:DNA complex to determine apparent affinity (K_D,app_). Each data point represents an average of at least three independent experiments ± the error determined using standard deviation of the mean.

### 2.6 Molecular dynamics simulations

Molecular dynamics (MD) simulations were performed using the coordinates from the X-ray crystallography structures of wild type APE1 in complex with various DNA. A total of 3 separate simulations were performed with APE1 bound to deoxy-AP product, ribo-AP product, and ribo-AP substrate DNA. Model preparation and simulations were performed using the AMBER v16 suite of programs for biomolecular simulations [27]. AMBER’s *ff14SB* [28] force-fields were used for the simulations, and the charges for the abasic nucleotides were generated based on the procedure described in the AMBER manual. The missing hydrogen atoms were added by AMBER’s *tleap* program. After processing the coordinates of the protein and substrate, all systems were neutralized by addition of counter-ions and the resulting system were solvated in a rectangular box of SPC/E water, with a 10 Å minimum distance between the protein and the edge of the periodic box. The prepared systems were equilibrated using a protocol described previously [29].

MD simulations were performed using NVIDIA graphical processing units (GPUs) and AMBER's *pmemd.cuda* simulation engine using our lab protocols published previously [30], [31]. The equilibrated systems were then used to run 1.0 μs of production MD under constant energy conditions (NVE ensemble). The use of NVE ensemble is preferred as it offers better computational stability and performance [32]. The production simulations were performed at a temperature of 300 K. As NVE ensemble was used for production runs, these values correspond to initial temperature at start of simulations. Temperature adjusting thermostat was not used in simulations; over the course of 1.0 μs simulations the temperature fluctuated around 300 K with RMS fluctuations between 2 and 4 K, which is typical for well equilibrated systems. A total of 10,000 conformational snapshots (stored every 100 ps) collected for each system was used for analysis. Analysis was performed using *ptraj* program included with AMBER. For extraction of the pucker angle, Eq. (2) was used [33]:

$$\tan P=\frac{\left(v_{4}+v_{1}\right)-\left(v_{3}+v_{0}\right)}{2v_{2}\left(\sin36^{{}^{\circ}}+\sin72^{{}^{\circ}}\right)}$$

where *P* represents the pseudorotation angle (°), *v* represents sugar torsion angles (°) with *v*0 = C4′-O4′-C1′–C2′, *v*1 = O4′-C1′–C2′–C3′, *v*2 = C1′–C2′–C3′-C4′, *v*3 = C2′–C3′-C4′-O4′, *v*4 = C3′-C4′-O4′-C1′. In cases where *v*2 is negative, 180° is added to the calculated *P* value.

### 2.7 Product formation assay

The relative cleavage activity of wild type APE1 on a rAP substrate was determined by qualitatively determining the amount of product formation for reactions with a fluorescein-containing rAP DNA substrate (same DNA substrate as used for kinetics). The reactions were carried out at 37 °C with 50 nM DNA substrate and 500 nM full length wild type APE1 in reaction buffer containing 50 mM HEPES (pH 7.5), 100 mM KCl, 0.1 mg/ml BSA, and either 5 mM MgCl_2_ or 100 mM EDTA. Using a rapid quench-flow (Kintek RQF-3), reactions were initiated by mixing DNA and APE1, followed by rapid quenching (with 200 mM EDTA) at a range of timepoints between 0.01 and 100 seconds. Quenched reactions were then diluted 1:1 with loading dye (100 mM EDTA, 80% deionized formamide, 0.25 mg/ml bromophenol blue, and 0.25 mg/ml xylene cyanol) and incubated at 95° for 6 min. Substrate and product DNA were separated on a 22% denaturing polyacrylamide gel and visualized (via the 5′-FAM label) using a Typhoon imager in fluorescence mode. All reactions were done in triplicate to ensure reproducibility and scientific rigor.

## 3 Results

### 3.1 Kinetic characterization of APE1 activity on abasic ribonucleotides

**Fig. 1:**
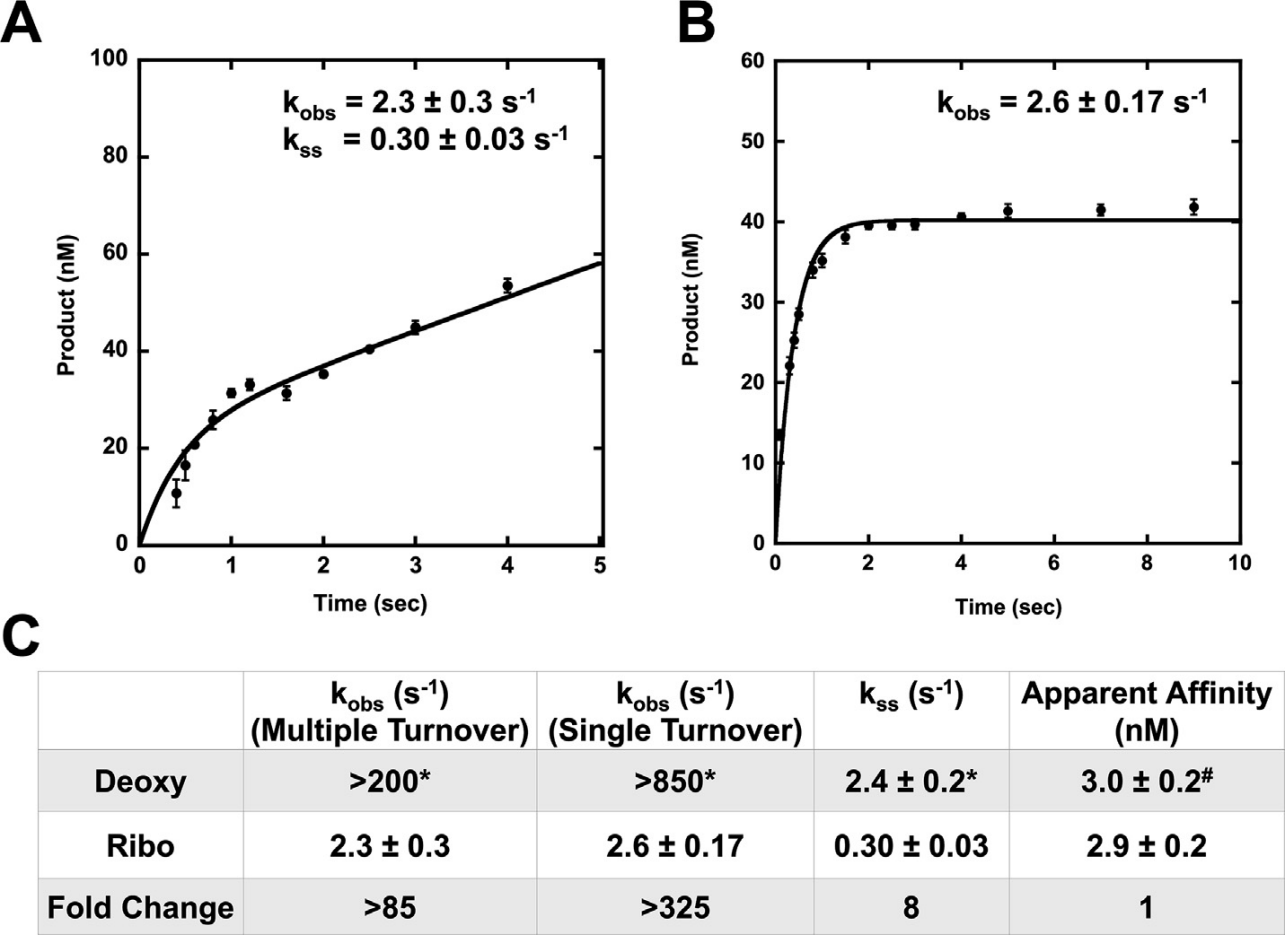
Pre-steady-state kinetic and binding characterization of wild-type APE1 with rAP substrate. (A) Multiple turnover and (B) single turnover kinetic time courses of product formation for the reaction with line of best fit (black line) and kinetic parameters. The time points are the mean and standard error of three independent experiments with error bars shown. Where error bars are not seen, they are present, but smaller than the data point. (C) Kinetic and binding parameters for wild-type APE1, with fold change comparing the deoxy- and ribo-AP site values. Previously published data represented by *[34]^#^[35]

To quantitatively analyze APE1 cleavage activity on an abasic ribonucleotide substrate, we employed pre-steady-state kinetics with a DNA oligonucleotide substrate containing a centrally located abasic ribonucleotide analog. Reactions contained an excess of DNA substrate which allowed for multiple turnovers of the cleavage reaction, generating a biphasic time course of product formation (Fig. 1A). This kinetic behavior shows that the first round of conversion of substrate to product occurs rapidly, but that some step following the chemical step is limiting the rate at which APE1 can dissociate from product and catalyze subsequent rounds of cleavage. This rate limiting step post-catalysis is presumably product release [34]. Thus, by fitting the data, the initial burst phase corresponds to the first enzymatic turnover and the rate of DNA cleavage (k_obs_), which is followed by a rate-limiting steady-state phase, presumably corresponding to the rate of product release (k_ss_). The k_obs_ and k_ss_ for WT APE1 with a rAP substrate are 2.3 ± 0.3 and 0.30 ± 0.03 sec^-1^, respectively (Fig. 1A). Additionally, since limiting the reaction to a single turnover can often provide a better estimation of k_obs_, we also preformed experiments under single turnover conditions which contained an excess of APE1. Consistent with the multiple turnover measurements, the single turnover k_obs_ is 2.6 ± 0.17 sec^-1^ (Fig. 1B). Importantly, this represents a substantial decrease in catalytic activity compared to the APE1 activity on a deoxy-AP site. Specifically, as the k_obs_ for APE1 on a deoxy-AP site has been reported to be > 200 s^−1^ (multiple turnover) and > 850 s^−1^ (single turnover), there is an > 85-fold and > 325-fold decrease in the observed burst rate, which for APE1 specifically describes the rate of phosphodiester bond cleavage (Fig. 1C)[34]. The k_ss_ values, which represent the steady-state rate-limiting step of the reaction, are more comparable, with only an 8-fold decrease in rate on a rAP site compared to a deoxy-AP site. This data suggests the reduction in activity for APE1 processing rAP substrates occurs predominantly at the catalysis step.

To account for any differences in APE1 DNA binding that may be contributing to the observed differences in the kinetics between rAP and deoxy-AP DNA substates, we probed the ability of WT APE1 to bind to the rAP DNA substrate. To this end, we utilized electrophoretic mobility shift assays (EMSA) to determine the apparent binding affinity (K_D, app_) for APE1: rAP complex formation. In this assay, the concentration of WT APE1 was varied in the presence of rAP DNA substrate and 1 mM EDTA to inhibit catalysis. This assay revealed that APE1 binds both deoxy and ribo abasic substrates with similar high affinities, producing K_D,app_ values of 3.0 ± 0.2 and 2.9 ± 0.2 nM, respectively (Fig. 1C, Supplementary Fig. 1)[35]. Together, this data highlights that APE1 efficiently engages the ribo abasic substrate and can turn it over, albeit at a reduced rate of cleavage.

### 3.2 Structural characterization of APE1 bound to a rAP site

To gain structural insight into the endoribonuclease activity of APE1, we collected X-ray crystallographic data of APE1 in complex with a 21-mer double-stranded DNA oligo containing a centrally located ribo abasic site analog. APE1 protein used for crystallography contains a truncation of the N-terminal 42 amino acids and the C138A mutation to aid in crystallization. We obtained crystals of both the substrate form (APE1 bound to an uncleaved rAP site) and product form (APE1 bound to a 5′ nicked rAP site), which diffracted to 2.05 and 2.10 Å, respectively (Supplementary Table 1). In the presence of MgCl_2_ APE1 readily crystallizes in the product form. However, in order to obtain substrate complex crystals, published APE1 structures utilize modifications (active site mutations or DNA modifications) to prevent catalysis. Here, a phosphorothioate backbone modification, where a sulfur is substituted for one of the non-bridging oxygens 5′ of the abasic site, was used in order to prevent catalysis and obtain substrate complex crystals [36], [37]. We compare our APE:rAP substrate structure to the previously published APE1:deoxy-AP substrate structure (PDB 5DFI), which was also obtained with a DNA substrate containing the phosphorothioate modification.

**Fig. 2:**
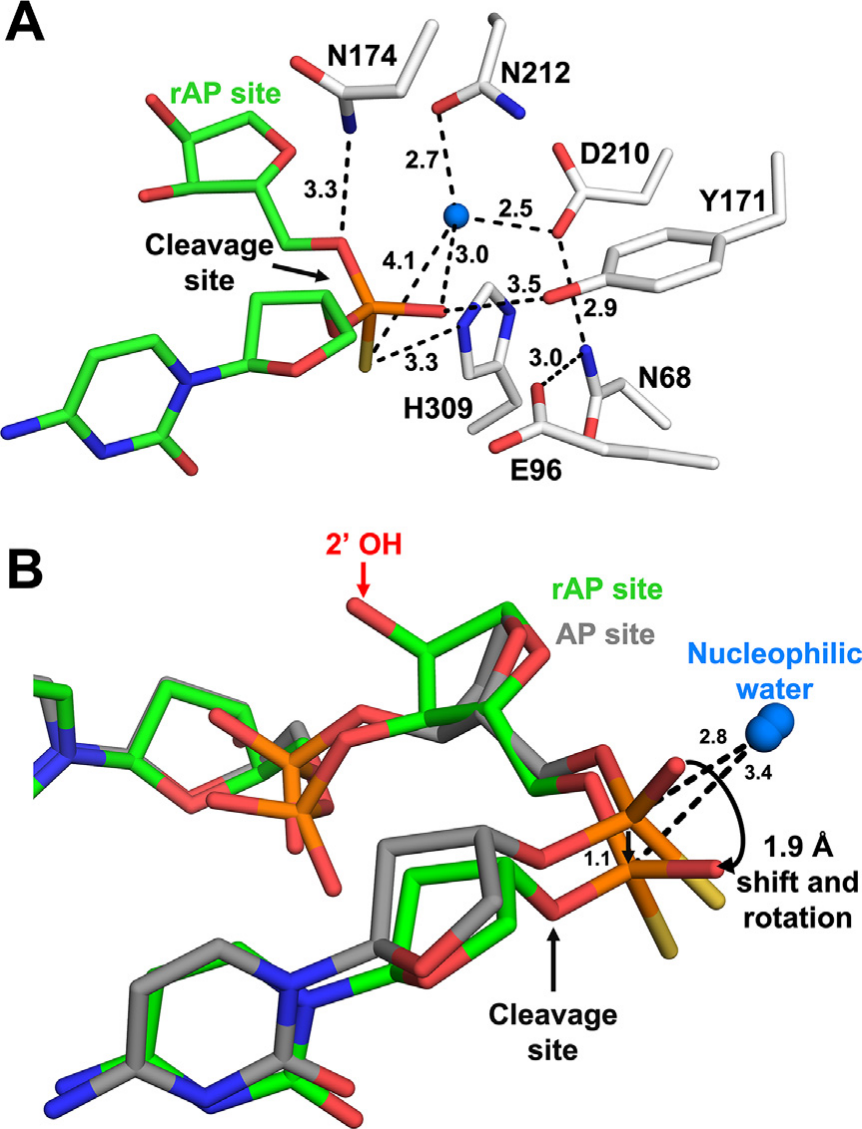
High-resolution structures of APE1: rAP substrate complex. (A) Focused view of the APE1 substrate complex active site. The DNA, including the rAP, are shown in stick format (green carbons). Active site waters are shown as blue spheres, with key residues (white sticks) and distances (Å) indicated. (B) Overlay of rAP (green) with previous structure for WT APE1 with deoxy-AP (grey, PDB 5DFI). (For interpretation of the references to color in this figure legend, the reader is referred to the web version of this article.)

The resulting APE1:rAP substrate complex reveals a ribo abasic site that is flipped out of the double-helix and into the APE1 active site, as seen previously for deoxy substrates [23], [38], [39]. The bridging backbone oxygen, situated 5′ to the abasic site, is near N174, while the non-bridging oxygen and sulfur are coordinated by Y171 and H309. An ordered water molecule is coordinated by the rAP site phosphate group, as well as the oxygen atoms of residues N212 and D210 (Fig. 2A). Interestingly, the 2′ OH of the ribose sugar is accommodated in the APE1 active site with no major clashes. The nearest protein contacts to the 2′ OH are A230 (backbone oxygen at 2.9 Å and side chain carbon at 3.9 Å) and W280 (side chain at 4.0 Å) (Supplemental Fig. 2A).

An overlay of the rAP substrate structure with the APE1 deoxy-AP substrate complex (PDB 5DFI), reveals notable changes in the conformational position of the sugar ring, specifically in the sugar pucker (Fig. 2B, see also Fig. 5A). Sugar pucker is defined by the non-planarity of the atoms that make up the five-membered sugar ring [33]. While deoxyribose nucleosides primarily adopt a C2′-endo form, the C3′-endo pucker places the hydroxyl substituents at the 2′ and 3′ positions further apart, and thus, is favored by ribonucleosides [40]. To determine if sugar puckers play a role in rAP cleavage by APE1, we determined the sugar pucker (pseudorotational angle, *P*) of the AP site for the deoxy and ribo AP:APE1 structures. This analysis revealed that the deoxy-AP substrate (PDB 5DFI) adopts a C2′-endo conformation (*P* = 173°), while the rAP is C3′-endo (*P* = 19°). In the rAP substrate structure, the altered sugar pucker of the rAP results in distortion of the phosphodiester backbone on both sides of the rAP site (Fig. 2B). Importantly, when comparing the deoxy-AP and ribo-AP substrate structures, the phosphate 5′ to the rAP is shifted by ~ 1.9 Å and rotated away from the nucleophilic water, and thus, is not ideally oriented for cleavage (Fig. 2B). In both structures, the nucleophilic water is positioned in the same location, but the shift observed for the rAP structure places the nucleophilic water further away from the phosphate backbone at 3.4 Å compared to 2.8 Å in the deoxy structure (Fig. 2B).

**Fig. 3:**
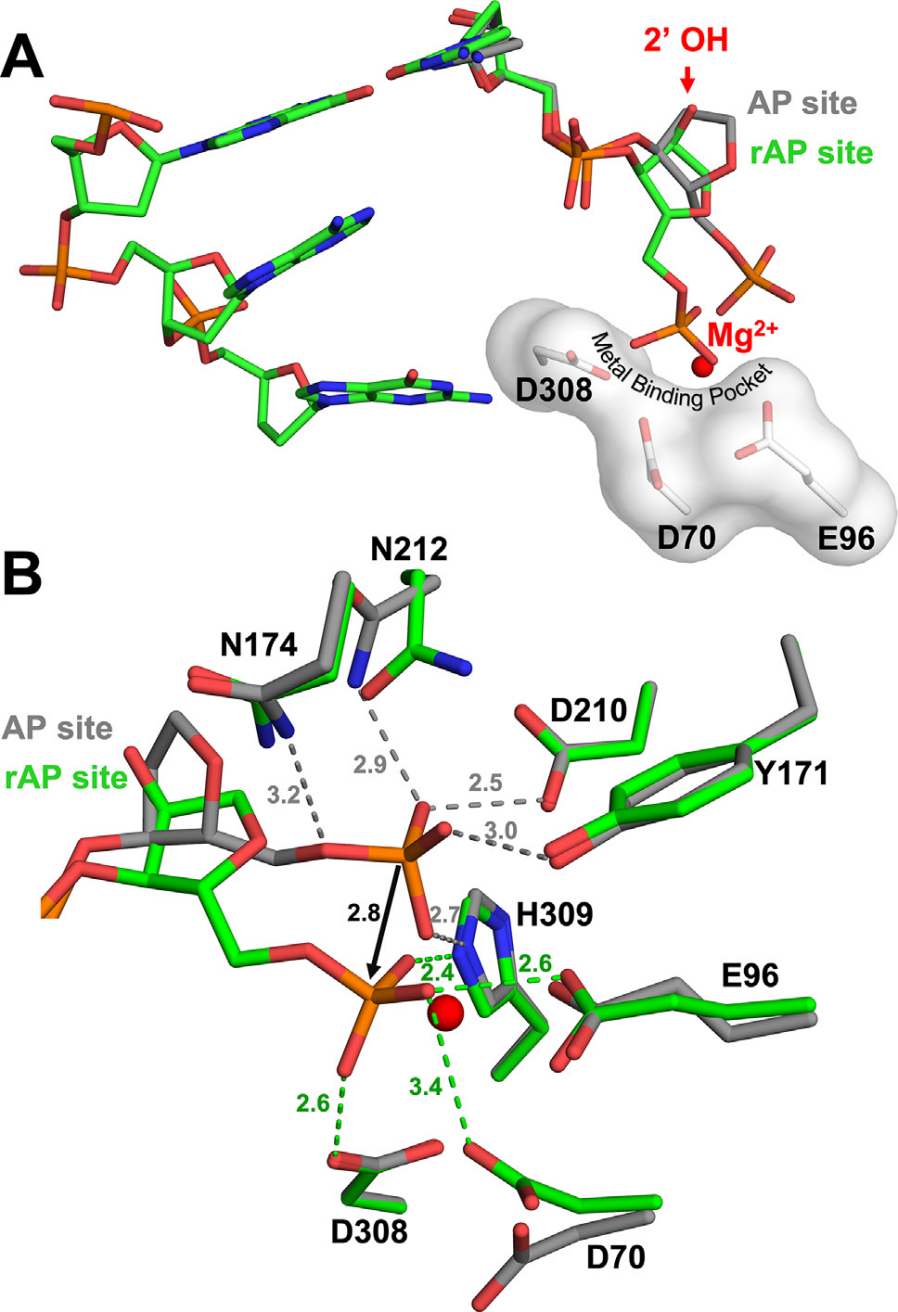
High-resolution structures of APE1: rAP product complex. (A) A focused view of the APE1 product complex active site. The rAP and flanking DNA are shown in stick format (green carbons). Metal binding pocket residues (white sticks) are indicated. (B) Overlay of rAP (green) with previous structure for WT APE1 with deoxy-AP (grey, PDB 5DFF). Key residues (shown as sticks) and distances (Å) are indicated. Mg^2+^ corresponds to the deoxy-AP structure and is represented by a red sphere. (For interpretation of the references to color in this figure legend, the reader is referred to the web version of this article.)

To generate product complex crystals, we rely on APE1 cleavage of the phosphodiester backbone to create a nick 5′ of the rAP site. In the resulting structure, we again see that the rAP site adopts the C3′-endo pucker (*P* = 16°), compared to the deoxy-AP site (PDB 5DFF) in C2′-endo (*P* = 185°). Similar to observations of the substrate structures, the rAP DNA backbone (Fig. 3, green) is shifted compared to the corresponding structure with deoxy-AP DNA (Fig. 3, grey), presumably due to the restrained sugar pucker of the rAP site. However, in the case of the product structure, there is also an accompanying 2.8 Å shift of the 5′ phosphate into the metal binding pocket (Fig. 3A and B). This reveals that the rAP is in the position normally occupied by a Mg^2+^. In the deoxy-AP product structure, the phosphate group is coordinated by active site residues N174, N212, D210, Y171 (Fig. 3B, grey). Interestingly, with the shift of the rAP phosphate backbone relative to the active site residues, these interactions are broken in the rAP product structure. Instead, the phosphate group oxygens are in position to interact with the residues that compose the metal binding pocket (E96, D70, D308) (Fig. 3B, green) [23], [39]. Importantly, with the phosphate in this pocket, it cannot simultaneously contain the catalytic metal, therefore there is no metal bound in this structure. The absence of metal in the binding pocket is an interesting finding as, in addition to the 200 mM MgCl_2_ in the crystal conditions, APE1 regularly purifies with Mg^2+^. Therefore, in APE1 product structures, this pocket is often occupied by Mg^2+^, suggesting that here the rAP is actually displacing the Mg^2+^ out or preventing it from efficiently binding to the metal binding pocket. This highlights the conflict between the rAP and binding of the catalytic metal. Together, these structures suggest that the reduced activity of the rAP substrate compared to a deoxy-AP substrate is likely due to the constrained sugar pucker of the rAP site, hindering ideal organization of the active site for catalysis.

### 3.3 Metal dependent cleavage of rAP by APE1

The observation that, in the rAP product structure, the catalytic metal and the phosphate group cannot simultaneously occupy the metal binding pocket (Fig. 3), led us to question the requirement of a catalytic metal during cleavage of a rAP site. Additionally, there are reports that APE1 has a metal independent cleavage activity on certain ribonucleotide substrates, as it is able to cleave *c-myc* RNA in the absence of any divalent metal ions [41], [42]. To test this, we utilized a product formation assay in the presence of 100 mM EDTA to chelate any contaminating metal. Our results show that with EDTA present, no product was generated over time, thus confirming that cleavage of an embedded abasic ribonucleotide by APE1 is a metal dependent activity (Supplemental Fig. 3).

**Fig. 4:**
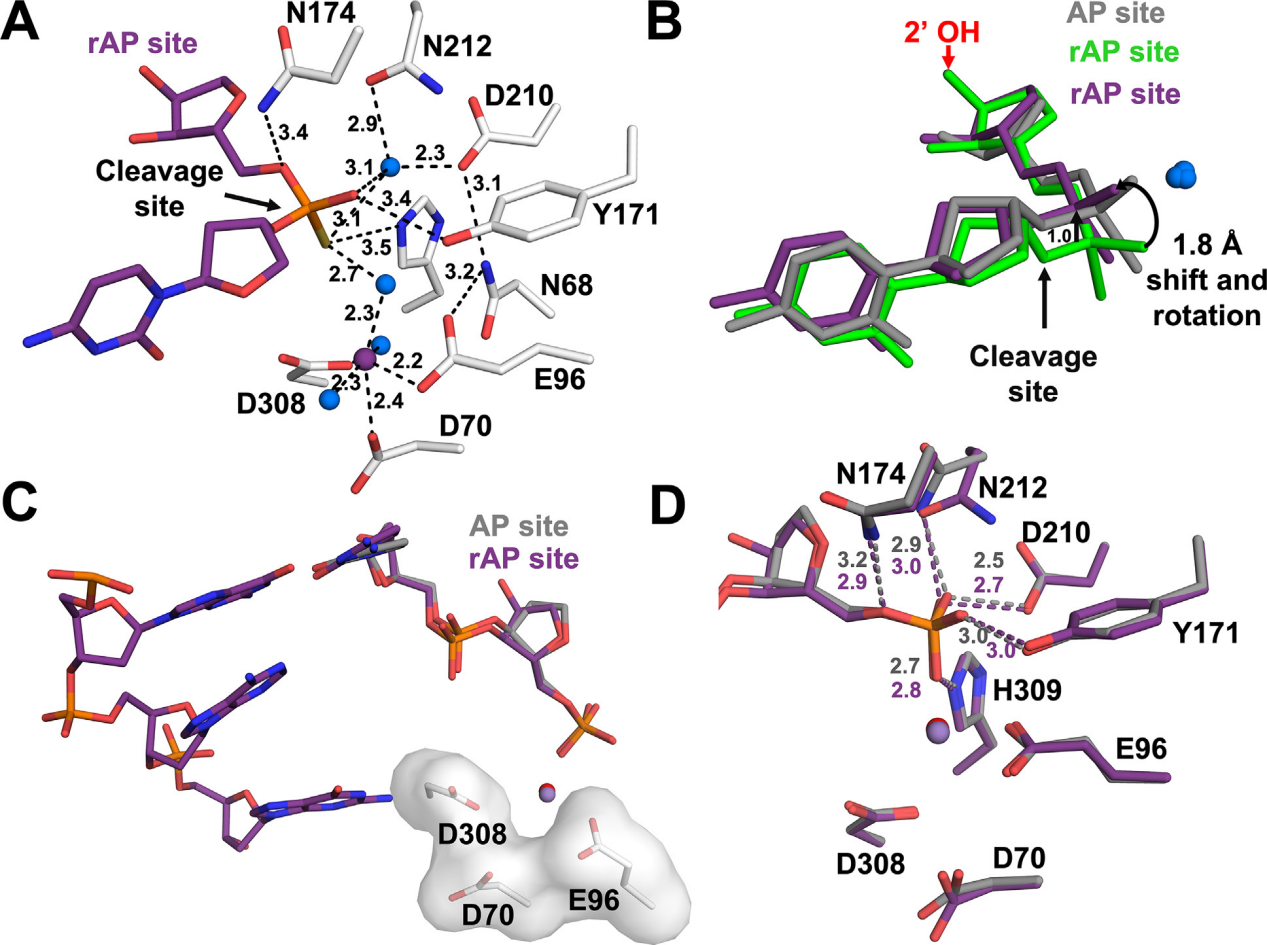
Metal-bound rAP structures. (A) A focused view of the APE1 substrate complex active site. The DNA, including the rAP, are shown in stick format (purple carbons). Active site waters and Mn^2+^ are shown as blue and purple spheres, respectively. Key residues (white sticks) and distances (Å) are indicated. (B) Overlay of rAP substrate (green) with previous structure for WT APE1 with deoxy-AP substrate (grey, PDB 5DFI), and Mn^2+^ bound rAP substrate (purple). (C) Overlay of rAP (purple) and deoxy (grey, PDB 5DFF) metal-bound product structures. (D) Key residues (shown as sticks) and distances (Å) are indicated. (For interpretation of the references to color in this figure legend, the reader is referred to the web version of this article.)

To further probe the role of the catalytic metal, we solved additional rAP:APE1 complex structures in the presence of MnCl_2_. Using this approach, we were able to obtain both substrate and product crystals with Mn^2+^ bound within the APE1 active site, which diffracted to 2.57 and 2.00 Å, respectively (Supplementary Table 1). One advantage of using Mn^2+^ in crystallography is that its position in the structure can be validated by identifying its anomalous signal. Similar to the deoxy-AP substrate structure (PDB 5DFI), in the rAP substrate structure the Mn^2+^ is coordinated directly by E96 , D70 , three water molecules, and D308 through a water-mediated interaction (Fig. 4A). An overlay of the two rAP structures reveals that in the Mn^2+^ bound rAP substrate active site, there is an accompanying 1.8 Å shift in the substrate backbone, which orients the phosphate group and non-bridging oxygens toward the nucleophilic water. This places the phosphate backbone in a position similar to the deoxy-AP site and poised for cleavage (Fig. 4B). This data suggests that a divalent metal is not only required for catalysis, but also assists in facilitating proper orientation of the DNA substrate in the APE1 active site for cleavage.

In the rAP product structure, Mn^2+^ sits in the metal binding pocket, overlaying with the Mg^2+^ of the deoxy-AP structure, and coordinated by E96, D70, D308 (Fig. 4C). Since both the Mn^2+^ metal ion and the 5′ phosphate cannot simultaneously occupy this pocket, the phosphate group of the rAP has shifted out of the pocket in the Mn^2+^ bound structure, where it now overlays with the position of 5′ phosphate in the deoxy-AP structure (Fig. 4C). This shift, compared to the non-metal bound structure, places the phosphate group in position to be coordinated by the same residues as the deoxy product (N174, N212, D210, Y171, H309) (Fig. 4D). In addition to this main conformation, a second conformation was identified in the Mn^2+^ bound rAP product structure at only 25% occupancy (Supplemental Fig. 4). In the second conformation, the rAP phosphate is shifted into the metal binding pocket, as seen in the non-metal bound product structure (Fig. 3), again highlighting the conflict that exists between the metal and the phosphate group. The existence of two alternate conformations of the nicked rAP site in this structure resulted in globular density that prevented accurate determination of the sugar pucker. Together, this data suggests that the APE1 catalytic metal helps to organize the APE1 active site during cleavage of an abasic ribonucleotide embedded in DNA. While few differences were seen when comparing the rAP metal-bound structures to deoxy-AP, this provides important insight into the fact that abasic ribonucleotides can be accommodated into the active site of APE1, which is essential for the RNA processing functions reported for APE1.

### 3.4 MD simulations of APE1 bound to a rAP

**Fig. 5:**
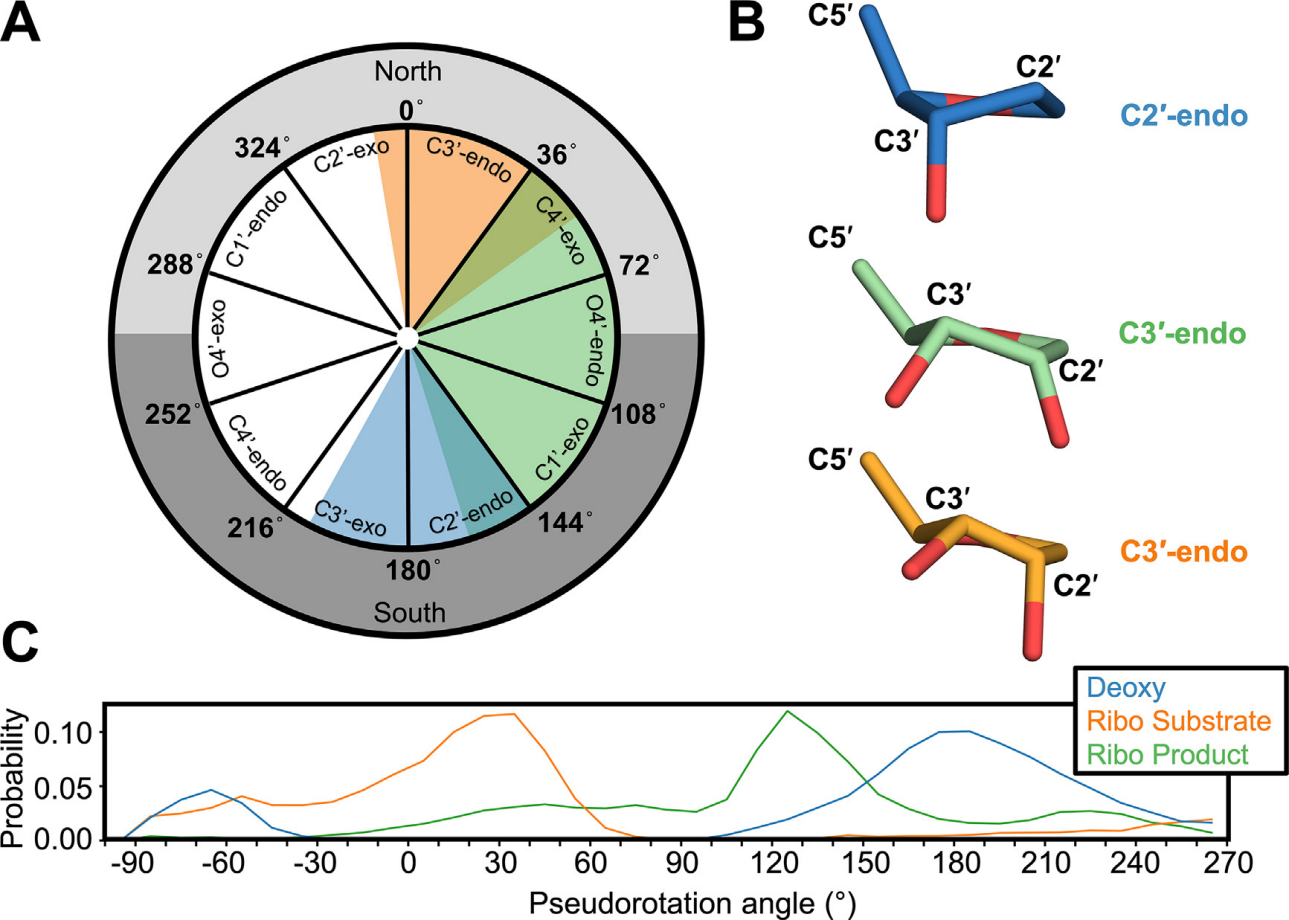
Sugar pucker analysis. (A) Pseudorotation wheel, adopted from Altona and Sundaralingam [33], each point on the circle represents a specific value of pseudorotation (P) as calculated with Eq. (2), and corresponding sugar pucker. General trends observed for MD data are super imposed on the wheel in the corresponding color from the key. (B) Abasic site of the deoxy-AP (PDB 5DFF, blue), rAP substrate (orange), and rAP product (green) crystal structures, highlighting position of C3′ and C2′ atoms and sugar pucker. (C) MD simulation probability of the pseudorotation angle for each of the abasic sites. (For interpretation of the references to color in this figure legend, the reader is referred to the web version of this article.)

To investigate the dynamics of the rAP substrate, we utilized MD simulations and further analyzed the conformation of the rAP sugar pucker. As implied by the pseudorotational wheel (Fig. 5A), sugar puckers are free to interconvert, and such rapid conversion has been observed in solution [40]. However, the relative populations of major sugar puckers are dependent on the attached substituent groups, which in this case includes the phosphate backbone and the 2′ OH (for the rAP) [33]. As observed in the crystal structure analysis, different sugar puckers were adopted by the deoxy and ribo-AP sites (Fig. 5B). Here, microsecond (1 μs) time-scale MD simulations were generated for both the substrate and product rAP structures, as well as the deoxy-AP product structure (PDB 5DFF, [39]). The MD data was then analyzed to determine the pseudorotational angle of the AP site throughout the simulation. In general, northern angles (-90 to + 90°), specifically near C3′-endo (-10 to + 40°), are associated with RNA, while southern angles (+90 to + 270°), specifically near C2′-endo (+140 to + 185°) are associated with B-DNA [40]. While it has been demonstrated that abasic sugars display a broadly distributed pseudorotational angle, that analysis was done in the absence of bound protein [43]. Here, we analyzed the sugar pucker dynamics of the abasic residues in the context of the APE1 active site. As seen previously for abasic sugars [43], our results reveal that both deoxy and ribo-AP sites display broadly distributed sugar puckers, but also highlight a clear distinction between the two. While the deoxy-AP site primarily occupies the C2′-endo and southern conformations, the rAP site in the substrate structure primarily occupies C3′-endo and northern conformations (Fig. 5C). Importantly, this is consistent with what was seen in our sugar pucker analysis of the X-ray crystal structures (Fig. 5B).

For additional insight, MD simulations were also completed with the free DNA (in the absence of the APE1 protein) for each of the abasic substrates (Supplemental Fig. 5). Similarly to observations in the APE1 active site, MD simulations show that both deoxy and ribo abasic sites adopt a widely distributed range of sugar puckers in free DNA. Our results show that both the ribo-AP substrate and product in free DNA adopt northern sugar puckers, while deoxy-AP adopts a southern pucker. While these puckers are not constrained to only C3′-endo and C2′-endo conformations, they are generally consistent with values that are fluctuating around the expected sugar puckers. Interestingly, we see that the free deoxy-AP DNA shows a surprisingly similar distribution as seen for the ribo-AP product DNA in the APE1 active site. Without the constraint of the DNA backbone being connected (since the product has a nicked backbone due to APE1 cleavage), the ribo-AP product is likely to be influenced by the APE1 active site more than its preferred sugar pucker. While this observed range of sugar puckers (about + 100°- +150°) may suggest rapid interconversion between the C2′-endo and C3′-endo pucker, it may also suggest that the APE1 active site is influencing the ribo-AP product into a more DNA-like position. Together, this data suggests that after cleavage occurs, the sugar pucker of the rAP site is less constrained, and the backbone has the freedom to be more dynamic in the APE1 active site.

## 4 Discussion

This study utilizes kinetic, structural, and computational methods to probe how APE1 processes rAP sites. Data presented here reveals that APE1 binds abasic ribonucleotides embedded in DNA with high affinity and cleaves with a moderately reduced activity compared to canonical deoxy-abasic site cleavage. Further structural analysis showed, as seen for ribonucleotides, that rAP sites in the APE1 active site prefer to adopt a C3′-endo sugar pucker. This C3′-endo sugar pucker is accommodated in the APE1 active site, but is not in ideal conformation for catalysis (i.e., cleavage by APE1). In this way, data suggests that the ribose sugar pucker influences its position in the active site and contributes to a decrease in cleavage activity compared to the deoxy-AP site. We showed that a divalent catalytic metal is not only required for this APE1 activity, but its presence helps to organize the active site for catalysis. In metal-bound structures, we observed that binding of the metal ion “pushes” the rAP into the proper orientation, highlighting the conflict that exists between the rAP site and the binding of the catalytic metal. Additionally, MD simulations further explored the dynamics of this mechanism, highlighting changes in the sugar pucker during APE1 cleavage.

### 4.1 APE1 nuclease activities

Human APE1 is an essential enzyme and multifunctional nuclease with AP-endonuclease, 3′ phosphodiesterase, 3′ to 5′ exonuclease, and RNA cleavage activities [6], [44], [45], [46], [47], [48], [49]. The data reported here allows for comparison of APE1 activity on rAP sites to its other known activities. We found that, as seen for other APE1 activities, APE1 has a fast rate of cleavage (k_obs_) and is then limited by a slow, presumably, product release step (k_ss_). Compared to canonical APE1 AP-endonuclease activity (with a deoxy abasic site), APE1 rAP activity has a reduced rate of cleavage by > 325-fold [34]. Furthermore, the steady-state turnover rate is less effected by the rAP, with only an 8-fold decrease. This is consistent with data published by Malfatti et al. [6], which reported that APE1 processes deoxy and ribo-AP with a similar efficiency using a steady-state kinetic analysis. Importantly, because our kinetics were performed under the pre-steady-state kinetic regime, we were able to differentiate the burst phase allowing determination of the rate for the chemistry step (k_obs_). This data reveals that APE1 cleaves rAP sites less efficiently than deoxy-AP sites, as is demonstrated by the reduction in the k_obs_ values. While these data show that rAP activity is slower than the exceedingly fast AP-endonuclease activity, the rAP kinetic rates are within the range of other known APE1 activities, as well as other DNA repair enzymes [21], [44], [50], [51], [52], [53], [54]. For example, rAP cleavage occurs at a similar rate to APE1 exonuclease activity, with only a 2.5-fold difference in catalytic rate between the two activities [21]. Additionally, a recent publication studied APE1 activity on an R-loop substrate consisting of an RNA-DNA hybrid with a single rAP site in the RNA strand. This RNA-DNA duplex substrate displayed a 32-fold decrease in catalysis (k_obs_) compared to our substrate with a single rAP site embedded in double-stranded DNA [11]. In the case of an RNA-DNA duplex, most of the APE1 footprint (nucleotides which are in contact or close proximity to the APE1 enzyme in the APE1-DNA complex) will consist of ribonucleotides, not just a single rAP to be accommodated in the active site. Additionally, having all RNA in the abasic strand would cause shifts from the B-form nucleic acid structure. Therefore, in addition to the change in position we observed for the ribo-AP in the APE1 active site, having a full strand of RNA bound to DNA is one explanation for the further reduction in kinetic activity for the RNA-DNA duplex substrate [11].

### 4.2 The APE1 active site readily accommodates rAP sites

The recognition of AP-DNA by APE1 is thought to be mediated by the unique structural flexibility of the target DNA substrate which allows APE1 to have an active role in DNA sculpting the AP-DNA in the active site [21], [23], [35], [39], [55]. Here, we discovered that sugar conformation, specifically sugar pucker, impacts APE1 activity by effecting the flexibility of the substrate and, thus, the DNA sculpting by APE1. One distinction between DNA and RNA is that deoxyribose nucleotides are primarily found in the C2′-endo form, while ribonucleotides favor a C3′-endo pucker to place the hydroxyl substituents at the 2′ and 3′ positions further apart [40]. Here, we studied the sugar pucker of a ribo-AP analog, tetrahydrofuran with an additional 2′ OH. Due to the chemical instability of the natural abasic site, they are historically difficult to study. As such, studying the sugar pucker of natural abasic sites has been reported to be challenging [56] and we believe our THF analog data provides insight into dynamics of the natural abasic site. We determined that in the APE1 active site there is balance between APE1 DNA sculpting and the preferred rAP site C3′-endo sugar pucker. Our structures reveal that the C3′-endo conformation of the rAP site is well accommodated within the APE1 active site. When a 2′ OH is modeled onto a C2′-endo abasic site, the 2′ OH clashes with side chain W280 (Supplemental Fig. 2B). Furthermore, with a shift into the preferred C3′-endo conformation, the rAP is accommodated in the active site without clashing with any protein residues (Supplemental Fig. 2C). While the C3′-endo sugar pucker avoids clashes in the active site, the added constraints hinder the ability of APE1 to sculpt the DNA into its ideal orientation in the active site. This likely contributes to the observed decrease in catalytic rate for the rAP substrate compared to deoxy-AP. These results highlight that APE1 did not evolve to have an amino acid preventing ribonucleotides from binding properly, such as a steric gate, as seen in many other enzymes and speaks to the biological importance of APE1 having activities on ribonucleotide substrates. In addition to cleaving genomic abasic ribonucleotides and R-loops, APE1 has also been reported to be involved in RNA metabolic and RNA-decay processes [41], [57], [58], [59], [60]. In all these functions, APE1 would have to accommodate ribonucleotides (which favor the C3′-endo sugar pucker) in its active site, and properly organize the active site for catalysis. Our data is consistent with the ability of APE1 to readily accommodate rAPs in all these contexts.

### 4.3 Biological implications for rAP processing

During replication of the human genome, replicative polymerases, Pol ε and Pol δ, are tasked with differentiating between deoxyribonucleotides and ribonucleotides to insert into the genomic DNA sequence. In part due to the great excess of cellular ribonucleotides over deoxyribonucleotides, polymerases will mistakenly incorporate ribonucleotides into the DNA sequence during replication [4]. Based on the ribonucleotide incorporation propensities for Pol ε and Pol δ, it is predicated that up to three million ribonucleotides may be incorporated during replication of the human genome [2], [3], [4]. Due to this prevalence, there is a repair pathway tasked with the removal of rNMPs, known as ribonucleotide excision repair (RER) pathway, which is initiated by RNase H2 cleavage [61], [62]. That said, with so many ribonucleotides incorporated into the genome that are susceptible to oxidative damage, it is likely there are also damaged rNMPs within the genome. For example, ribonucleotide abasic sites could be generated by either spontaneous hydrolysis of the RNA N-glycosidic bond or by glycosylase cleavage of oxidized rNMPs [11], [63]. While the RNA glycosidic bond is stronger and, thus, less susceptible to spontaneous hydrolysis than DNA, even a small fraction of incorporated ribonucleotides becoming abasic could pose a threat to genomic stability if not repaired by APE1 [6]. In addition, RNA abasic sites are formed early in RNA synthesis, as they were detected in nascent RNA and coupled to R-loops, which form during transcription [11]. Based on the known stability of R-loops and rAP sites, it is likely that these R-loops with rAP sites are very stable and could influence the expression of that transcript. In addition to transcriptional regulation, RNA abasic sites may exist as an RNA repair intermediate, removing misincorporated or damaged ribonucleotides, but also may have a regulatory function through altering the RNA shape, stability, and protein interactions [11]. In all of these scenarios APE1 plays a key role in processing rAP sites to protect genomic stability.

In addition to APE1 initially processing rAP sites, we must also consider the importance of downstream processing. In base excision repair, after APE1 activity, the DNA is further processed by the lyase and then polymerase activity of polymerase β. Currently, it is unknown if the lyase activity of polymerase β is active on a ribonucleotide substrate. Additionally, the handoff between APE1 and polymerase β is of interest in the field, as the DNA is thought to be channeled from one enzyme to the next to protect the repair intermediates. Here, we report that APE1 has a slow turnover step, which may allow APE1 to remain bound to the rAP nicked product for handoff to the next processing enzyme. Our structural insights highlighting the importance of sugar pucker will likely affect both downstream processing and substrate channeling of APE1′s ribonucleotide substrates. Additional studies will be needed to further understand these complex mechanisms.

## CRediT authorship contribution statement

**Nicole M. Hoitsma:** Conceptualization, Investigation, Formal analysis, Validation, Writing - original draft, Writing - review & editing. **Timothy H. Click:** Investigation, Formal analysis, Software. **Pratul K. Agarwal:** Investigation, Formal analysis, Software, Writing - review & editing. **Bret D. Freudenthal:** Conceptualization, Writing - review & editing, Supervision, Funding acquisition.

## Declaration of Competing Interest

The authors declare that they have no known competing financial interests or personal relationships that could have appeared to influence the work reported in this paper.

## References

[1] Traut T.W. (1994). Physiological concentrations of purines and pyrimidines. Mol Cell Biochem.

[2] Goksenin A.Y. (2012). Human DNA polymerase epsilon is able to efficiently extend from multiple consecutive ribonucleotides. J Biol Chem.

[3] Clausen A.R. (2013). Ribonucleotide incorporation, proofreading and bypass by human DNA polymerase delta. DNA Repair (Amst).

[4] Williams J.S., Lujan S.A., Kunkel T.A. (2016). Processing ribonucleotides incorporated during eukaryotic DNA replication. Nat Rev Mol Cell Biol.

[5] Reijns M.M., Rabe B., Rigby R., Mill P., Astell K., Lettice L. (2012). Enzymatic removal of ribonucleotides from DNA is essential for mammalian genome integrity and development. Cell.

[6] Malfatti MC. et al., Abasic and oxidized ribonucleotides embedded in DNA are processed by human APE1 and not by RNase H2. Nucleic Acids Res, 2017. 45(19): p. 11193-11212. 10.1093/nar/gkx723 PMC5737539 28977421

[7] Whitaker A.M., et al. (2017). Base excision repair of oxidative DNA damage: from mechanism to disease. Front Biosci (Landmark Ed).

[8] Wallace S.S. (2014). Base excision repair: a critical player in many games. DNA Repair (Amst).

[9] Wilson D.M., Bohr V.A. (2007). The mechanics of base excision repair, and its relationship to aging and disease. DNA Repair (Amst).

[10] Beard W.A., Horton J.K., Prasad R., Wilson S.H. (2019). Eukaryotic Base Excision Repair: New Approaches Shine Light on Mechanism. Annu Rev Biochem.

[11] Liu Y., Rodriguez Y., Ross R.L., Zhao R., Watts J.A., Grunseich C. (2020). RNA abasic sites in yeast and human cells. Proc Natl Acad Sci U S A.

[12] Crossley M.P., Bocek M., Cimprich K.A. (2019). R-Loops as Cellular Regulators and Genomic Threats. Mol Cell.

[13] Niehrs C., Luke B. (2020). Regulatory R-loops as facilitators of gene expression and genome stability. Nat Rev Mol Cell Biol.

[14] Skourti-Stathaki K., Proudfoot N.J. (2014). A double-edged sword: R loops as threats to genome integrity and powerful regulators of gene expression. Genes Dev.

[15] Roberts R., Crothers D. (1992). Stability and properties of double and triple helices: dramatic effects of RNA or DNA backbone composition. Science.

[16] Kupfer P.A., Leumann C.J. (2007). The chemical stability of abasic RNA compared to abasic DNA. Nucleic Acids Res.

[17] Castellano-Pozo M., Santos-Pereira José M., Rondón A., Barroso S., Andújar E., Pérez-Alegre M. (2013). R loops are linked to histone H3 S10 phosphorylation and chromatin condensation. Mol Cell.

[18] Chen P.B., Chen H.V., Acharya D., Rando O.J., Fazzio T.G. (2015). R loops regulate promoter-proximal chromatin architecture and cellular differentiation. Nat Struct Mol Biol.

[19] Grunseich C., Wang I.X., Watts J.A., Burdick J.T., Guber R.D., Zhu Z. (2018). Senataxin Mutation Reveals How R-Loops Promote Transcription by Blocking DNA Methylation at Gene Promoters. Mol Cell.

[20] Sollier J., Cimprich K.A. (2015). Breaking bad: R-loops and genome integrity. Trends Cell Biol.

[21] Whitaker A.M., Flynn T.S., Freudenthal B.D. (2018). Molecular snapshots of APE1 proofreading mismatches and removing DNA damage. Nat Commun.

[22] He H., Chen Q., Georgiadis M.M. (2014). High-resolution crystal structures reveal plasticity in the metal binding site of apurinic/apyrimidinic endonuclease I. Biochemistry.

[23] Mol C.D. (2000). DNA-bound structures and mutants reveal abasic DNA binding by APE1 and DNA repair coordination [corrected]. Nature.

[24] Adams P.D., Afonine P.V., Bunkóczi G., Chen V.B., Davis I.W., Echols N. (2010). PHENIX: a comprehensive Python-based system for macromolecular structure solution. Acta Crystallogr D Biol Crystallogr.

[25] Emsley P., Cowtan K. (2004). Coot: model-building tools for molecular graphics. Acta Crystallogr D Biol Crystallogr.

[26] Vascotto C., Fantini D., Romanello M., Cesaratto L., Deganuto M., Leonardi A. (2009). APE1/Ref-1 interacts with NPM1 within nucleoli and plays a role in the rRNA quality control process. Mol Cell Biol.

[27] Case DA. et al., AMBER 15. University of California, San Francisco., 2015.

[28] Maier J.A., Martinez C., Kasavajhala K., Wickstrom L., Hauser K.E., Simmerling C. (2015). ff14SB: improving the accuracy of protein side chain and backbone parameters from ff99SB. J Chem Theory Comput.

[29] Ramanathan A., Agarwal P.K., Petsko G.A. (2011). Evolutionarily Conserved Linkage between Enzyme Fold, Flexibility, and Catalysis. PLoS Biol.

[30] Ramanathan A., Agarwal P.K., Kurnikova M., Langmead C.J. (2010). An online approach for mining collective behaviors from molecular dynamics simulations. J Comput Biol.

[31] Duff M.R., Borreguero J.M., Cuneo M.J., Ramanathan A., He J., Kamath G. (2018). Modulating Enzyme Activity by Altering Protein Dynamics with Solvent. Biochemistry.

[32] Beck D.A., Daggett V. (2004). Methods for molecular dynamics simulations of protein folding/unfolding in solution. Methods.

[33] Altona C., Sundaralingam M. (1972). Conformational analysis of the sugar ring in nucleosides and nucleotides. A new description using the concept of pseudorotation. J Am Chem Soc.

[34] Maher R.L., Bloom L.B. (2007). Pre-steady-state kinetic characterization of the AP endonuclease activity of human AP endonuclease 1. J Biol Chem.

[35] Hoitsma N.M. (2020). AP-endonuclease 1 sculpts DNA through an anchoring tyrosine residue on the DNA intercalating loop. Nucleic Acids Res.

[36] Mundle S.T., Delaney J.C., Essigmann J.M., Strauss P.R. (2009). Enzymatic mechanism of human apurinic/apyrimidinic endonuclease against a THF AP site model substrate. Biochemistry.

[37] Wilson D.M., Takeshita M., Grollman A.P., Demple B. (1995). Incision activity of human apurinic endonuclease (Ape) at abasic site analogs in DNA. J Biol Chem.

[38] Fairlamb M.S., Whitaker A.M., Freudenthal B.D. (2018). Apurinic/apyrimidinic (AP) endonuclease 1 processing of AP sites with 5' mismatches. Acta Crystallogr D Struct Biol.

[39] Freudenthal B.D., Beard W.A., Cuneo M.J., Dyrkheeva N.S., Wilson S.H. (2015). Capturing snapshots of APE1 processing DNA damage. Nat Struct Mol Biol.

[40] Neidle S. Principles of nucleic acid structure. 1st ed. 2008: Elsevier; Academic Press. Ch 2: p. 20-37.

[41] Kim W.C., King D., Lee C.H. (2010). RNA-cleaving properties of human apurinic/apyrimidinic endonuclease 1 (APE1). Int J Biochem Mol Biol.

[42] Kuznetsova A.A., Gavrilova A.A., Novopashina D.S., Fedorova O.S., Kuznetsov N.A. (2021). Mutational and Kinetic Analysis of APE1 Endoribonuclease Activity. Mol Biol.

[43] Barsky D., Foloppe N., Ahmadia S., Wilson III D.M., MacKerell A.D. (2000). New insights into the structure of abasic DNA from molecular dynamics simulations. Nucleic Acids Res.

[44] Li M., Wilson D.M. (2014). Human apurinic/apyrimidinic endonuclease 1. Antioxid Redox Signal.

[45] Whitaker A.M., Freudenthal B.D. (2018). APE1: A skilled nucleic acid surgeon. DNA Repair (Amst).

[46] Tell G., Quadrifoglio F., Tiribelli C., Kelley M.R. (2009). The many functions of APE1/Ref-1: not only a DNA repair enzyme. Antioxid Redox Signal.

[47] Wong D., DeMott M.S., Demple B. (2003). Modulation of the 3'–>5'-exonuclease activity of human apurinic endonuclease (Ape1) by its 5'-incised Abasic DNA product. J Biol Chem.

[48] Chen D.S., Herman T., Demple B. (1991). Two distinct human DNA diesterases that hydrolyze 3'-blocking deoxyribose fragments from oxidized DNA. Nucleic Acids Res.

[49] Dyrkheeva N.S., Lomzov A.A., Pyshnyi D.V., Khodyreva S.N., Lavrik O.I. (2006). Efficiency of exonucleolytic action of apurinic/apyrimidinic endonuclease 1 towards matched and mismatched dNMP at the 3' terminus of different oligomeric DNA structures correlates with thermal stability of DNA duplexes. Biochim Biophys Acta.

[50] Schermerhorn K.M., Delaney S. (2014). A chemical and kinetic perspective on base excision repair of DNA. Acc Chem Res.

[51] Tumbale P., Williams J.S., Schellenberg M.J., Kunkel T.A., Williams R.S. (2014). Aprataxin resolves adenylated RNA-DNA junctions to maintain genome integrity. Nature.

[52] Comeaux E.Q., Cuya S.M., Kojima K., Jafari N., Wanzeck K.C., Mobley J.A. (2015). Tyrosyl-DNA phosphodiesterase I catalytic mutants reveal an alternative nucleophile that can catalyze substrate cleavage. J Biol Chem.

[53] Burkovics P., Hajdu I., Szukacsov V., Unk I., Haracska L. (2009). Role of PCNA-dependent stimulation of 3'-phosphodiesterase and 3'-5' exonuclease activities of human Ape2 in repair of oxidative DNA damage. Nucleic Acids Res.

[54] Wiederhold L., Leppard J.B., Kedar P., Karimi-Busheri F., Rasouli-Nia A., Weinfeld M. (2004). AP endonuclease-independent DNA base excision repair in human cells. Mol Cell.

[55] Tsutakawa S.E., Lafrance-Vanasse J., Tainer J.A. (2014). The cutting edges in DNA repair, licensing, and fidelity: DNA and RNA repair nucleases sculpt DNA to measure twice, cut once. DNA Repair (Amst).

[56] Chen J. et al., DNA oligonucleotides with A, T, G or C opposite an abasic site: structure and dynamics. Nucleic Acids Res, 2008. 36(1): p. 253-62. 10.1093/nar/gkm622 PMC2248740 18025040

[57] Barnes T., Kim W.-C., Mantha A.K., Kim S.-E., Izumi T., Mitra S. (2009). Identification of Apurinic/apyrimidinic endonuclease 1 (APE1) as the endoribonuclease that cleaves c-myc mRNA. Nucleic Acids Res.

[58] Berquist B.R., McNeill D.R., Wilson D.M. (2008). Characterization of abasic endonuclease activity of human Ape1 on alternative substrates, as well as effects of ATP and sequence context on AP site incision. J Mol Biol.

[59] Antoniali G., Malfatti M.C., Tell G. (2017). Unveiling the non-repair face of the Base Excision Repair pathway in RNA processing: A missing link between DNA repair and gene expression?. DNA Repair (Amst).

[60] Kim W.-C., Berquist B.R., Chohan M., Uy C., Wilson D.M., Lee C.H. (2011). Characterization of the endoribonuclease active site of human apurinic/apyrimidinic endonuclease 1. J Mol Biol.

[61] Burgers P.M.J., Kunkel T.A. (2017). Eukaryotic DNA Replication Fork. Annu Rev Biochem.

[62] Sparks J., Chon H., Cerritelli S., Kunkel T., Johansson E., Crouch R. (2012). RNase H2-initiated ribonucleotide excision repair. Mol Cell.

[63] Lenz S.A.P., Wetmore S.D. (2016). Evaluating the Substrate Selectivity of Alkyladenine DNA Glycosylase: The Synergistic Interplay of Active Site Flexibility and Water Reorganization. Biochemistry.

