# Supplementary material for: Altered APE1 activity on abasic ribonucleotides is mediated by changes in the nucleoside sugar pucker
Source: Comput Struct Biotechnol J. 2021 May 25;19:3293–302. doi: 10.1016/j.csbj.2021.05.035 (PMC8207216; doi:10.1016/j.csbj.2021.05.035)
Supplement: Supplementary data 1 [file mmc1.pdf]

Supplementary Information for

Altered APE1 activity on abasic ribonucleotides is mediated by changes in the nucleoside sugar pucker

Nicole M. Hoitsma[a], Timothy H. Click[b], Pratul K. Agarwal[b], and Bret D. Freudenthal[a]*

[a] Department of Biochemistry and Molecular Biology, University of Kansas Medical Center, Kansas City, KS 66160, USA

[b] Department of Physiological Sciences and High-Performance Computing Center, Oklahoma State University, Stillwater, OK 74078, USA

* To whom correspondence should be addressed. Tel: 913-588-5560; Email: bfreudenthal@kumc.edu

Supplementary Figure 1
Supplementary Figure 2
Supplementary Figure 3
Supplementary Figure 4
Supplementary Figure 5
Supplementary Table 1

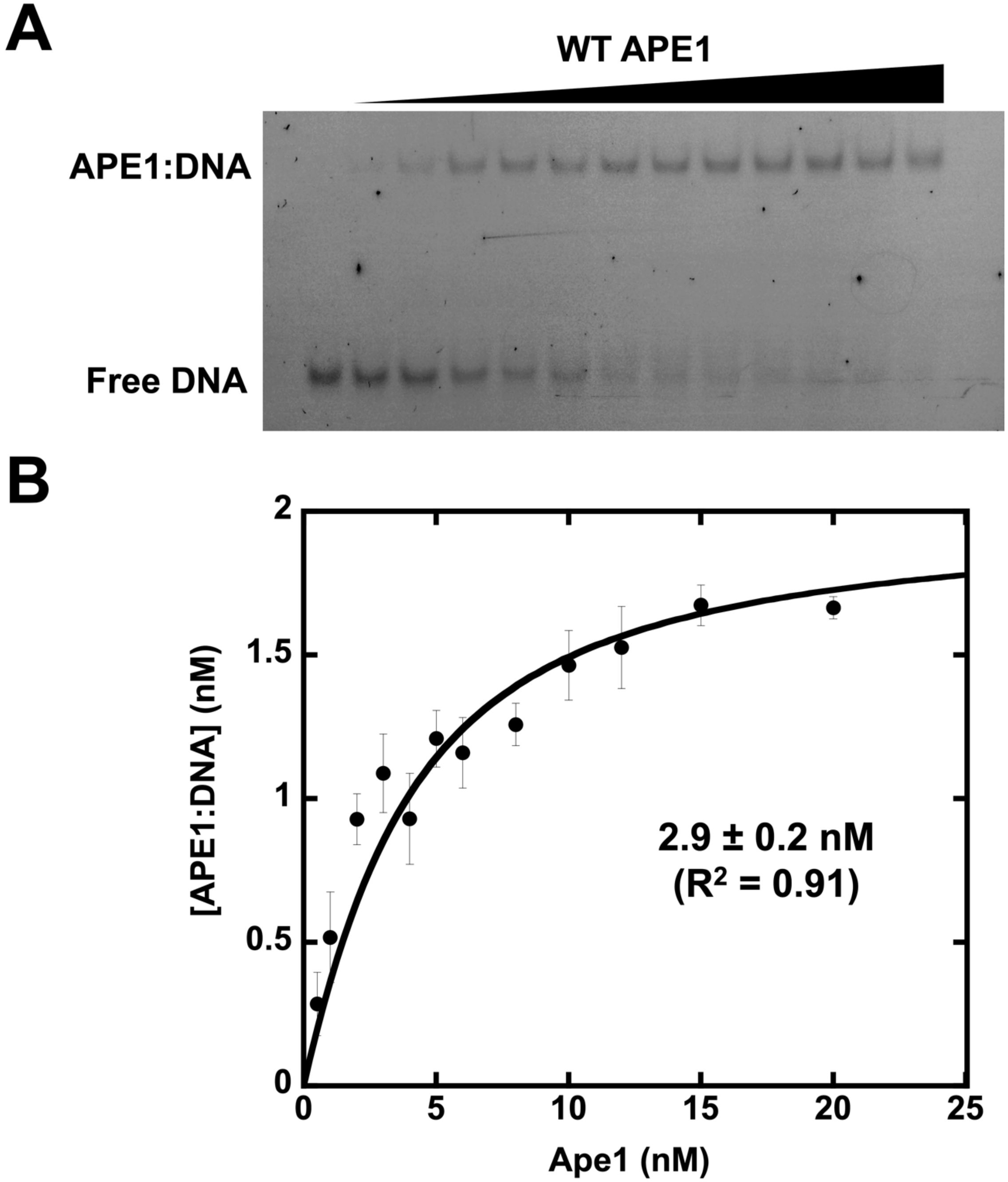

Supplementary Figure 1. Ribo AP-site binding analysis. (A) Representative EMSA gel image for WT APE1 binding. (B) Quantification of EMSA analysis where the line represents best fit to equation 1 (see Methods). Curve for deoxy-abasic site binding via EMSA analysis can be found within reference 35.

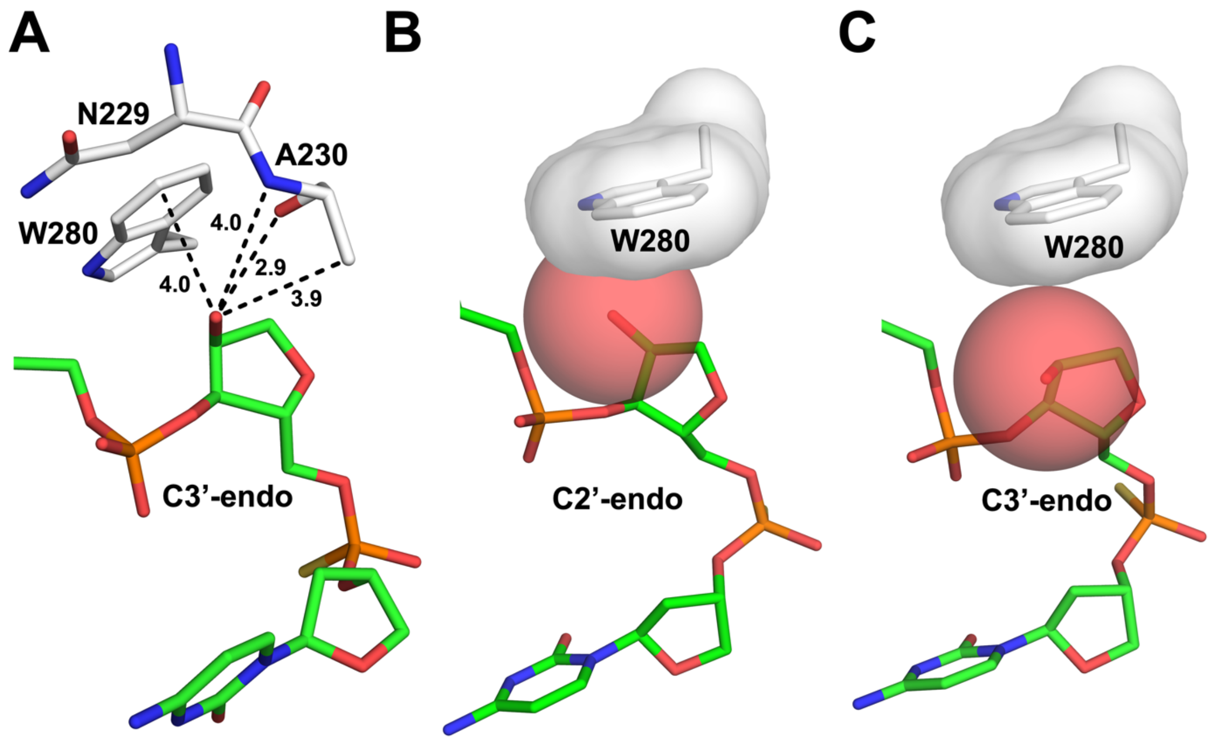

Supplementary Figure 2. Active site accommodation of the ribose 2´ OH. (A) C3´-endo conformation rAP site with all contacts ≤ 4.0 Å and key resides (white) shown as sticks. (B) C2´-endo conformation abasic site (PDB 5DFF) with additional 2´ OH modeled (red sphere represents oxygen van der waals radius). (C) C3´-endo conformation rAP site (red sphere represents oxygen van der waals radius).

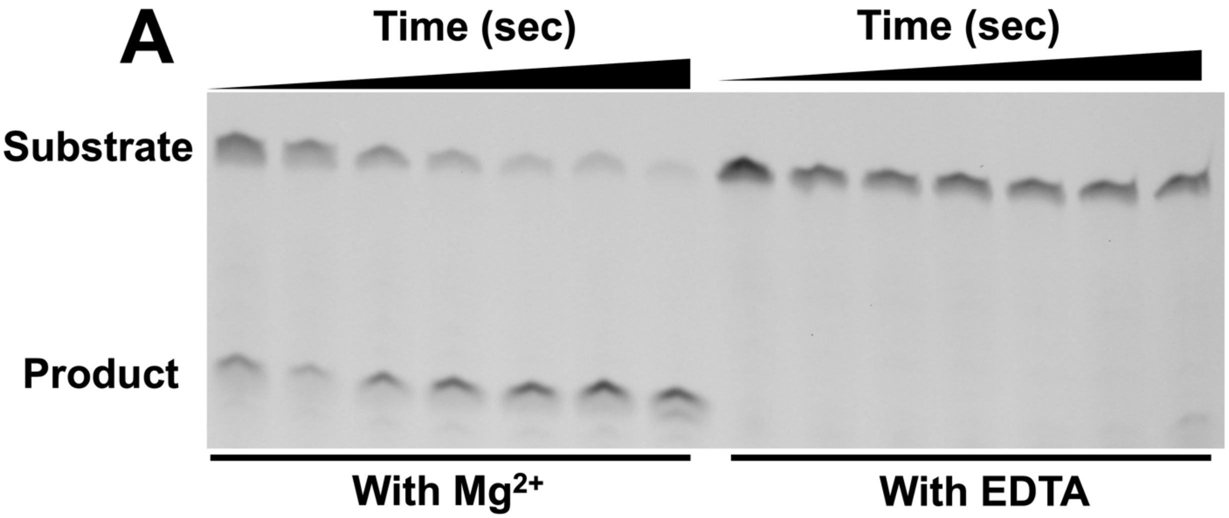

Supplementary Figure 3. Divalent Metal Dependence. (A) Representative gel showing separation of APE1 substrate and product bands from reactions completed in the presence of either Mg^{2+} or EDTA.

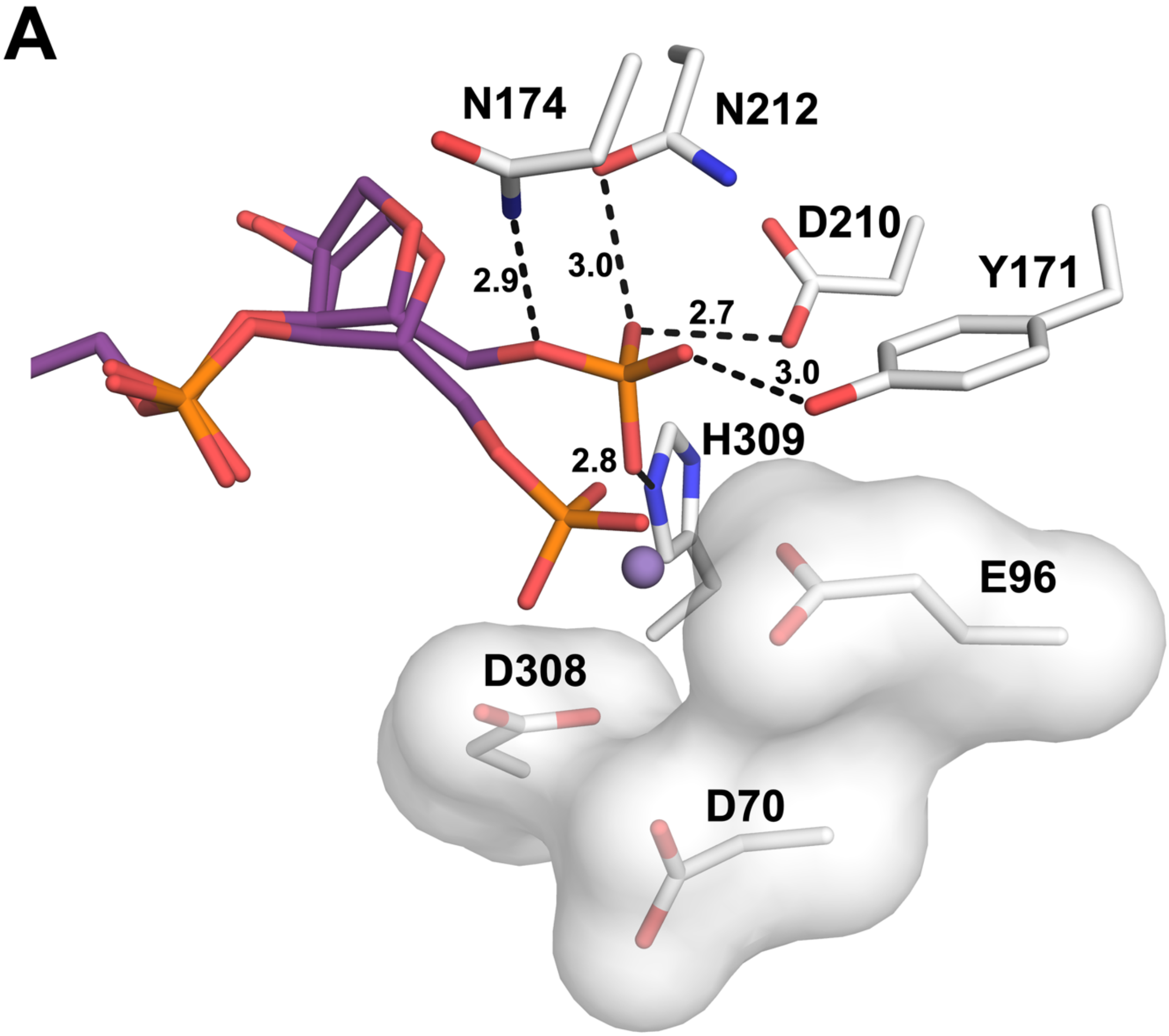

Supplementary Figure 4. Metal-bound rAP product structure alternative conformation. (A) A focused view of the APE1 Mn-bound product complex active site. Conformation A (75% occupancy) is shown interacting with key residues (white sticks) with distances (Å) shown. Conformation B (25% occupancy) is shown in metal binding pocket (residues shown with surface representation).

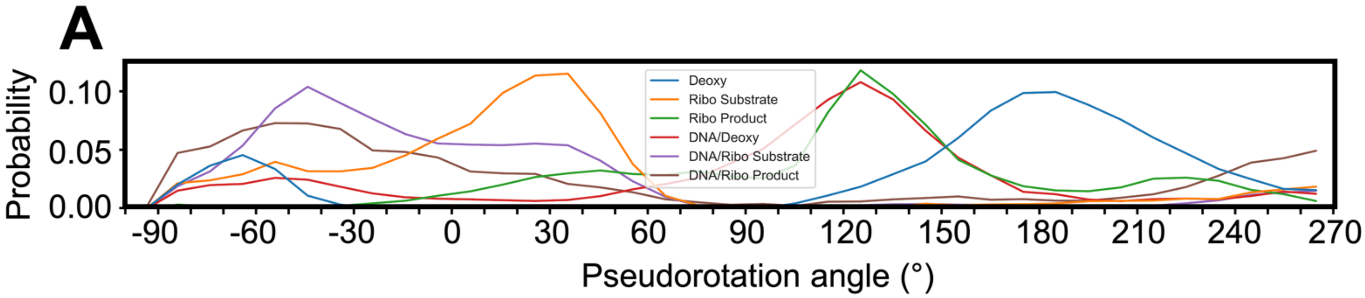

Supplementary Figure 5. MD simulations for APE1-bound and free DNA substrates. (A) MD simulation probability of the pseudorotation angle for each of the abasic sites. Free DNA substrates denoted as DNA/.

Supplementary Table 1. Data collection and refinement statistics of APE1: rAP co-complexes. Each structure was obtained from a single crystal. Values in parentheses are for highest-resolution shell.

	rAP Product	rAP Product with Mn	rAP Substrate	rAP Substrate with Mn
**Data collection**				
Space group	P 1	P 1	P 1	P 1
Cell dimensions				
a, *b*, *c* (Å)	44.1,61.1,73.0	44.2,61.6,73.0	44.2,60.9,73.2	44.2,60.9,73.3
α, β, γ (°)	83.7,78.1,87.5	83.7,78.1,87.4	83.6,78.0,87.1	82.9,77.6,86.2
Resolution (Å)	25 – 2.08	25 – 1.99	25 – 2.05	50 – 2.56
R_{meas}[a] (%)	0.119 (0.723)	0.080 (0.574)	0.106 (0.000)	0.146 (0.000)
$I/\sigma I$	12.5 (1.8)	21.7 (2.2)	12.3 (1.3)	12.3 (1.4)
cc1/2[b]	(0.680)	(0.789)	(0.505)	(0.632)
Completeness[a] (%)	99.5 (97.9)	96.4 (88.5)	99.2 (95.3)	99.8 (97.7)
Redundancy[a]	3.6 (3.3)	5.6 (3.3)	3.9 (3.6)	5.6 (4.4)
**Refinement**				
Resolution (Å)	23.69 – 2.08	24.72 – 1.99	24.46 – 2.05	48.82 – 2.56
No. reflections	75243	66383	57272	31441
$R_{work/}$ R_{free}	21.9/26.3	19.2/23.7	19.7/24.5	18.3/24.8
No. atoms				
Protein	4324	4332	4319	4367
DNA	835	895	835	835
Water	239	325	190	47
B-factors (Å^2)				
Protein	43.90	30.58	34.00	36.95
DNA	61.46	48.81	54.04	54.22
Water	44.57	33.36	33.21	32.56
R.m.s deviations				
Bond length (Å)	0.009	0.010	0.012	0.010
Bond angles (º)	1.110	1.264	1.248	1.307
PDB ID	7LPG	7LPH	7LPI	7LPJ
