# Supplementary material for: Altered APE1 activity on abasic ribonucleotides is mediated by changes in the nucleoside sugar pucker
Source: Comput Struct Biotechnol J. 2021 May 25;19:3293–302. doi: 10.1016/j.csbj.2021.05.035 (PMC8207216; doi:10.1016/j.csbj.2021.05.035)
Supplement: Supplementary data 3 [file mmc3.zip › 7LPH.pdf]

Full wwPDB X-ray Structure Validation Report (i)

Feb 17, 2021 – 05:06 PM EST

PDB ID	:	7LPH
Title	:	APE1 Mn-bound product complex with abasic ribonucleotide DNA
Deposited on	:	2021-02-11
Resolution	:	1.99 Å(reported)

This is a Full wwPDB X-ray Structure Validation Report.

This report is produced by the wwPDB biocuration pipeline after annotation of the structure.

We welcome your comments at *validation@mail.wwpdb.org*
A user guide is available at
https://www.wwpdb.org/validation/2017/XrayValidationReportHelp
with specific help available everywhere you see the (i) symbol.

CONFIDENTIAL VALIDATION REPORT

The following versions of software and data (see references (i)) were used in the production of this report:

MolProbity	:	4.02b-467
Xtriage (Phenix)	:	1.13
EDS	:	2.17.1
Percentile statistics	:	20191225.v01 (using entries in the PDB archive December 25th 2019)
Refmac	:	5.8.0158
CCP4	:	7.0.044 (Gargrove)
Ideal geometry (proteins)	:	Engh & Huber (2001)
Ideal geometry (DNA, RNA)	:	Parkinson et al. (1996)
Validation Pipeline (wwPDB-VP)	:	2.17.1

1 Overall quality at a glance (i)

The following experimental techniques were used to determine the structure:
X-RAY DIFFRACTION

The reported resolution of this entry is 1.99 Å.

Percentile scores (ranging between 0-100) for global validation metrics of the entry are shown in the following graphic. The table shows the number of entries on which the scores are based.

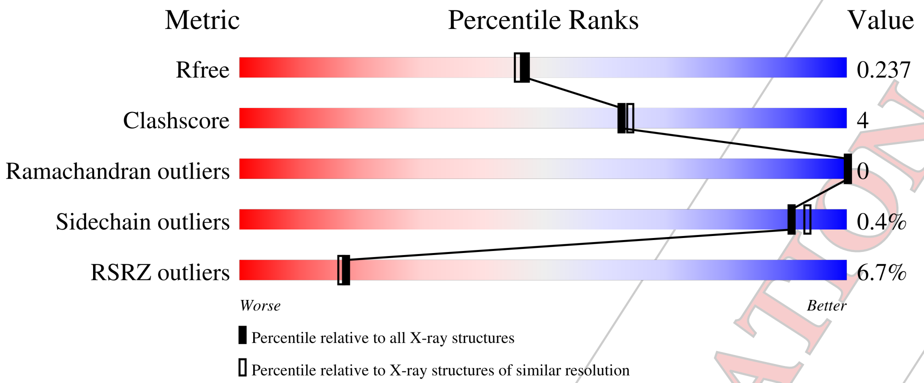

Metric	Whole archive (#Entries)	Similar resolution (#Entries, resolution range(Å))
R_{free}	130704	8085 (2.00-2.00)
Clashscore	141614	9178 (2.00-2.00)
Ramachandran outliers	138981	9054 (2.00-2.00)
Sidechain outliers	138945	9053 (2.00-2.00)
RSRZ outliers	127900	7900 (2.00-2.00)

The table below summarises the geometric issues observed across the polymeric chains and their fit to the electron density. The red, orange, yellow and green segments of the lower bar indicate the fraction of residues that contain outliers for >=3, 2, 1 and 0 types of geometric quality criteria respectively. A grey segment represents the fraction of residues that are not modelled. The numeric value for each fraction is indicated below the corresponding segment, with a dot representing fractions <=5% The upper red bar (where present) indicates the fraction of residues that have poor fit to the electron density. The numeric value is given above the bar.

Mol	Chain	Length	Quality of chain
1	B	276	9% / 91% 8% •
1	D	276	4% / 92% 6% •
2	C	21	10% / 57% 43%
3	E	10	90% 10%
4	A	11	18% / 73% 27%

CONFIDENTIAL VALIDATION REPORT

2 Entry composition (i)

There are 6 unique types of molecules in this entry. The entry contains 5579 atoms, of which 0 are hydrogens and 0 are deuteriums.

In the tables below, the ZeroOcc column contains the number of atoms modelled with zero occupancy, the AltConf column contains the number of residues with at least one atom in alternate conformation and the Trace column contains the number of residues modelled with at most 2 atoms.

- Molecule 1 is a protein called DNA-(apurinic or apyrimidinic site) lyase.

Mol	Chain	Residues	Atoms					ZeroOcc	AltConf	Trace
			Total	C	N	O	S			
1	B	273	2166	1386	374	398	8	0	0	0
1	D	271	2166	1387	375	395	9	0	2	0

There are 2 discrepancies between the modelled and reference sequences:

Chain	Residue	Modelled	Actual	Comment	Reference
B	138	ALA	CYS	engineered mutation	UNP P27695
D	138	ALA	CYS	engineered mutation	UNP P27695

- Molecule 2 is a DNA chain called DNA (5'-D(*GP*GP*AP*TP*CP*CP*GP*TP*CP*GP*AP*GP*CP*GP*CP*AP*TP*CP*AP*GP*C)-3').

Mol	Chain	Residues	Atoms					ZeroOcc	AltConf	Trace
			Total	C	N	O	P			
2	C	21	428	203	82	123	20	0	0	0

- Molecule 3 is a DNA chain called DNA (5'-D(*GP*CP*TP*GP*AP*TP*GP*CP*GP*C)-3 ').

Mol	Chain	Residues	Atoms					ZeroOcc	AltConf	Trace
			Total	C	N	O	P			
3	E	10	244	116	46	71	11	0	2	0

- Molecule 4 is a DNA chain called DNA (5'-R(P*N)-D(P*CP*GP*AP*CP*GP*GP*AP*TP*CP*C)-3').

Mol	Chain	Residues	Atoms					ZeroOcc	AltConf	Trace
			Total	C	N	O	P			
4	A	11	249	115	42	79	13	0	2	0

CONFIDENTIAL VALIDATION REPORT

- Molecule 5 is MANGANESE (II) ION (three-letter code: MN) (formula: Mn).

Mol	Chain	Residues	Atoms	ZeroOcc	AltConf
5	D	1	Total Mn<br>1 1	0	1

- Molecule 6 is water.

Mol	Chain	Residues	Atoms	ZeroOcc	AltConf
6	B	87	Total O<br>87 87	0	0
6	C	20	Total O<br>20 20	0	0
6	D	202	Total O<br>202 202	0	4
6	E	8	Total O<br>8 8	0	1
6	A	8	Total O<br>8 8	0	0

CONFIDENTIAL VALIDATION REPORT

3 Residue-property plots (i)

These plots are drawn for all protein, RNA, DNA and oligosaccharide chains in the entry. The first graphic for a chain summarises the proportions of the various outlier classes displayed in the second graphic. The second graphic shows the sequence view annotated by issues in geometry and electron density. Residues are color-coded according to the number of geometric quality criteria for which they contain at least one outlier: green = 0, yellow = 1, orange = 2 and red = 3 or more. A red dot above a residue indicates a poor fit to the electron density (RSRZ > 2). Stretches of 2 or more consecutive residues without any outlier are shown as a green connector. Residues present in the sample, but not in the model, are shown in grey.

- Molecule 1: DNA-(apurinic or apyrimidinic site) lyase

Chain B:

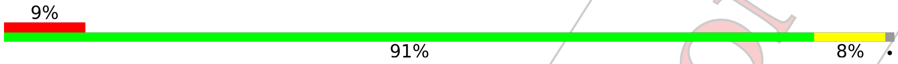

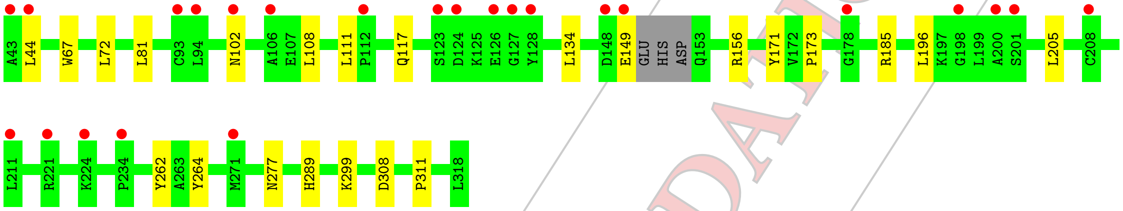

- Molecule 1: DNA-(apurinic or apyrimidinic site) lyase

Chain D:

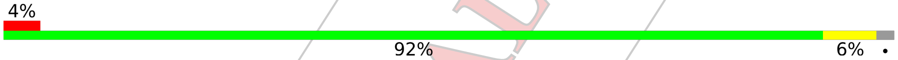

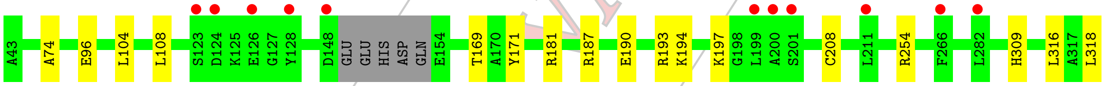

- Molecule 2: DNA (5'-D(*GP*GP*AP*TP*CP*CP*GP*TP*CP*GP*AP*GP*CP*GP*CP*AP *TP*CP*AP*GP*C)-3')

Chain C:

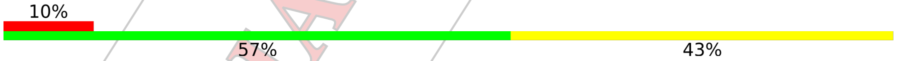

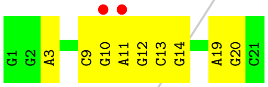

- Molecule 3: DNA (5'-D(*GP*CP*TP*GP*AP*TP*GP*CP*GP*C)-3')

Chain E:

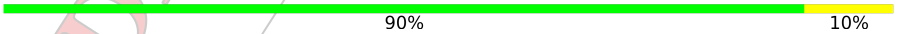

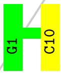

- Molecule 4: DNA (5'-R(P*N)-D(P*CP*GP*AP*CP*GP*GP*AP*TP*CP*C)-3')

Chain A:

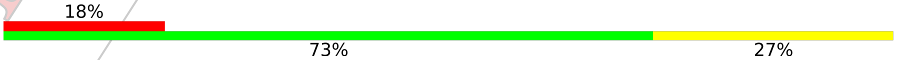

CONFIDENTIAL VALIDATION REPORT

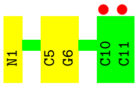

CONFIDENTIAL VALIDATION REPORT

4 Data and refinement statistics (i)

Property	Value	Source
Space group	P 1	Depositor
Cell constants<br>a, b, c, α, β, γ	44.25Å 61.57Å 72.96Å<br>83.65° 78.15° 87.42°	Depositor
Resolution (Å)	24.72 – 1.99<br>24.72 – 1.99	Depositor<br>EDS
% Data completeness<br>(in resolution range)	65.0 (24.72-1.99)<br>66.5 (24.72-1.99)	Depositor<br>EDS
R_{merge}	(Not available)	Depositor
R_{sym}	(Not available)	Depositor
$< I/\sigma(I) >$ [1]	2.37 (at 1.99Å)	Xtriage
Refinement program	PHENIX 1.17.1_3660	Depositor
R, R_{free}	0.192 , 0.237<br>0.192 , 0.237	Depositor<br>DCC
R_{free} test set	1511 reflections (4.20%)	wwPDB-VP
Wilson B-factor (Å^2)	22.2	Xtriage
Anisotropy	0.060	Xtriage
Bulk solvent k_{sol}(e/Å^3), B_{sol}(Å^2)	0.34 , 48.3	EDS
L-test for twinning[2]	$< \|L\| > = 0.50$, $< L^2 > = 0.33$	Xtriage
Estimated twinning fraction	No twinning to report.	Xtriage
F_o,F_c correlation	0.94	EDS
Total number of atoms	5579	wwPDB-VP
Average B, all atoms (Å^2)	33.0	wwPDB-VP

Xtriage's analysis on translational NCS is as follows: *The largest off-origin peak in the Patterson function is 5.77% of the height of the origin peak. No significant pseudotranslation is detected.*

[1] Intensities estimated from amplitudes.

[2] Theoretical values of $< |L| >$, $< L^2 >$ for acentric reflections are 0.5, 0.333 respectively for untwinned datasets, and 0.375, 0.2 for perfectly twinned datasets.

CONFIDENTIAL VALIDATION REPORT

5 Model quality (i)

5.1 Standard geometry (i)

Bond lengths and bond angles in the following residue types are not validated in this section: MN

The Z score for a bond length (or angle) is the number of standard deviations the observed value is removed from the expected value. A bond length (or angle) with $|Z| > 5$ is considered an outlier worth inspection. RMSZ is the root-mean-square of all Z scores of the bond lengths (or angles).

Mol	Chain	Bond lengths		Bond angles	
		RMSZ	#|Z| >5	RMSZ	#|Z| >5
1	B	0.47	0/2222	0.69	0/3012
1	D	0.48	0/2223	0.66	0/3013
2	C	0.77	0/480	0.96	0/739
3	E	0.99	0/272	1.12	0/416
4	A	0.73	0/249	0.94	0/381
All	All	0.56	0/5446	0.76	0/7561

There are no bond length outliers.

There are no bond angle outliers.

There are no chirality outliers.

There are no planarity outliers.

5.2 Too-close contacts (i)

In the following table, the Non-H and H(model) columns list the number of non-hydrogen atoms and hydrogen atoms in the chain respectively. The H(added) column lists the number of hydrogen atoms added and optimized by MolProbity. The Clashes column lists the number of clashes within the asymmetric unit, whereas Symm-Clashes lists symmetry-related clashes.

Mol	Chain	Non-H	H(model)	H(added)	Clashes	Symm-Clashes
1	B	2166	0	2145	19	0
1	D	2166	0	2145	12	0
2	C	428	0	236	7	0
3	E	244	0	136	1	0
4	A	249	0	139	7	0
5	D	1	0	0	0	0
6	A	8	0	0	0	0
6	B	87	0	0	3	0

Continued on next page...

CONFIDENTIAL VALIDATION REPORT

Continued from previous page...

Mol	Chain	Non-H	H(model)	H(added)	Clashes	Symm-Clashes
6	C	20	0	0	2	0
6	D	202	0	0	7	0
6	E	8	0	0	1	0
All	All	5579	0	4801	43	0

The all-atom clashscore is defined as the number of clashes found per 1000 atoms (including hydrogen atoms). The all-atom clashscore for this structure is 4.

All (43) close contacts within the same asymmetric unit are listed below, sorted by their clash magnitude.

Atom-1	Atom-2	Interatomic distance (Å)	Clash overlap (Å)
2:C:12:DG:H2”	2:C:13:DC:H5”	1.79	0.65
6:D:501:HOH:O	4:A:1[A]:N:H1’	1.97	0.64
2:C:19:DA:H2”	2:C:20:DG:H5”	1.81	0.63
6:D:501:HOH:O	4:A:1[B]:N:C1’	2.50	0.58
6:D:501:HOH:O	4:A:1[A]:N:C1’	2.50	0.57
1:D:193:ARG:HD3	6:D:521:HOH:O	2.05	0.57
1:B:44:LEU:HD23	1:B:277:ASN:CB	2.35	0.56
1:B:67:TRP:CD2	1:B:311:PRO:HG3	2.41	0.55
1:D:309:HIS:NE2	4:A:1[B]:N:OP1	2.26	0.54
1:B:81:LEU:HD22	1:B:111:LEU:HD11	1.91	0.52
1:D:96:GLU:HG3	1:D:171:TYR:CZ	2.46	0.51
4:A:5:DC:H2”	4:A:6:DG:C8	2.46	0.51
1:B:44:LEU:HD23	1:B:277:ASN:HB2	1.92	0.51
6:D:501:HOH:O	4:A:1[B]:N:H1’	2.11	0.50
1:B:149:GLU:HG2	1:B:149:GLU:O	2.10	0.50
1:B:111:LEU:C	6:B:408:HOH:O	2.50	0.49
1:B:117:GLN:HG2	1:B:134:LEU:HG	1.94	0.48
2:C:10:DG:H5’	6:C:105:HOH:O	2.12	0.48
1:B:262:TYR:HA	1:B:264:TYR:CZ	2.49	0.47
1:D:316:LEU:HB3	1:D:318:LEU:HD13	1.95	0.47
1:B:44:LEU:HD23	1:B:277:ASN:HB3	1.97	0.47
1:D:169:THR:HA	1:D:208:CYS:O	2.14	0.46
1:B:67:TRP:CE3	1:B:311:PRO:HG3	2.50	0.46
1:D:254:ARG:HD3	1:D:254:ARG:HA	1.56	0.44
2:C:10:DG:H1’	2:C:11:DA:H5’	1.99	0.43
2:C:14:DG:H3’	1:D:74:ALA:HB2	2.01	0.43
1:D:187:ARG:NH1	6:D:503:HOH:O	2.30	0.43
1:B:196:LEU:HD22	1:B:205:LEU:HD21	2.00	0.43
1:B:44:LEU:CD2	1:B:277:ASN:HB2	2.48	0.43
1:B:299:LYS:HA	1:B:299:LYS:HD2	1.88	0.42

Continued on next page...

CONFIDENTIAL VALIDATION REPORT

Continued from previous page...

Atom-1	Atom-2	Interatomic distance (Å)	Clash overlap (Å)
1:B:111:LEU:CA	6:B:408:HOH:O	2.66	0.42
1:B:308:ASP:OD2	1:B:308:ASP:N	2.53	0.42
1:D:104:LEU:HB3	1:D:108:LEU:HD12	2.02	0.42
1:B:289:HIS:HB2	6:B:472:HOH:O	2.20	0.41
1:B:72:LEU:HD21	1:B:108:LEU:HD11	2.03	0.41
1:B:156:ARG:HD3	1:B:171:TYR:O	2.20	0.41
1:D:181:ARG:NE	6:D:517:HOH:O	2.50	0.41
3:E:10[A]:DC:P	6:E:101:HOH:O	2.78	0.41
1:B:173:PRO:O	1:B:185:ARG:HD3	2.21	0.40
1:D:96:GLU:OE1	4:A:1[B]:N:OP3	2.38	0.40
2:C:3:DA:N3	6:C:102:HOH:O	2.37	0.40
2:C:9:DC:H2”	2:C:10:DG:C8	2.56	0.40
1:D:190:GLU:HG2	1:D:194:LYS:HE3	2.03	0.40

There are no symmetry-related clashes.

5.3 Torsion angles (i)

5.3.1 Protein backbone (i)

In the following table, the Percentiles column shows the percent Ramachandran outliers of the chain as a percentile score with respect to all X-ray entries followed by that with respect to entries of similar resolution.

The Analysed column shows the number of residues for which the backbone conformation was analysed, and the total number of residues.

Mol	Chain	Analysed	Favoured	Allowed	Outliers	Percentiles	
1	B	269/276 (98%)	256 (95%)	13 (5%)	0	100	100
1	D	269/276 (98%)	264 (98%)	5 (2%)	0	100	100
All	All	538/552 (98%)	520 (97%)	18 (3%)	0	100	100

There are no Ramachandran outliers to report.

5.3.2 Protein sidechains (i)

In the following table, the Percentiles column shows the percent sidechain outliers of the chain as a percentile score with respect to all X-ray entries followed by that with respect to entries of similar resolution.

CONFIDENTIAL VALIDATION REPORT

The Analysed column shows the number of residues for which the sidechain conformation was analysed, and the total number of residues.

Mol	Chain	Analysed	Rotameric	Outliers	Percentiles	
1	B	231/235 (98%)	230 (100%)	1 (0%)	91	93
1	D	231/235 (98%)	230 (100%)	1 (0%)	91	93
All	All	462/470 (98%)	460 (100%)	2 (0%)	91	93

All (2) residues with a non-rotameric sidechain are listed below:

Mol	Chain	Res	Type
1	B	102	ASN
1	D	197	LYS

Sometimes sidechains can be flipped to improve hydrogen bonding and reduce clashes. There are no such sidechains identified.

5.3.3 RNA (i)

There are no RNA molecules in this entry.

5.4 Non-standard residues in protein, DNA, RNA chains (i)

There are no non-standard protein/DNA/RNA residues in this entry.

5.5 Carbohydrates (i)

There are no monosaccharides in this entry.

5.6 Ligand geometry (i)

Of 1 ligands modelled in this entry, 1 is monoatomic - leaving 0 for Mogul analysis.

There are no bond length outliers.

There are no bond angle outliers.

There are no chirality outliers.

There are no torsion outliers.

There are no ring outliers.

No monomer is involved in short contacts.

CONFIDENTIAL VALIDATION REPORT

5.7 Other polymers ⓘ

There are no such residues in this entry.

5.8 Polymer linkage issues ⓘ

There are no chain breaks in this entry.

CONFIDENTIAL VALIDATION REPORT

6 Fit of model and data (i)

6.1 Protein, DNA and RNA chains (i)

In the following table, the column labelled '#RSRZ> 2' contains the number (and percentage) of RSRZ outliers, followed by percent RSRZ outliers for the chain as percentile scores relative to all X-ray entries and entries of similar resolution. The OWAB column contains the minimum, median, 95th percentile and maximum values of the occupancy-weighted average B-factor per residue. The column labelled 'Q< 0.9' lists the number of (and percentage) of residues with an average occupancy less than 0.9.

Mol	Chain	Analysed	<RSRZ>	#RSRZ>2			OWAB(Å^2)	Q<0.9
1	B	273/276 (98%)	0.60	24 (8%)	10	9	19, 34, 59, 82	0
1	D	271/276 (98%)	0.22	11 (4%)	37	36	12, 22, 47, 77	0
2	C	21/21 (100%)	0.59	2 (9%)	8	7	30, 47, 62, 83	0
3	E	10/10 (100%)	0.31	0	100	100	41, 45, 48, 48	0
4	A	10/11 (90%)	0.99	2 (20%)	1	0	32, 55, 75, 82	0
All	All	585/594 (98%)	0.43	39 (6%)	17	17	12, 29, 58, 83	0

All (39) RSRZ outliers are listed below:

Mol	Chain	Res	Type	RSRZ
1	B	127	GLY	6.7
1	B	126	GLU	6.5
1	D	201	SER	6.2
4	A	11	DC	5.3
1	B	124	ASP	5.2
1	B	128	TYR	4.8
1	D	128	TYR	4.8
1	D	124	ASP	4.7
1	D	126	GLU	4.5
1	B	149	GLU	4.4
1	B	44	LEU	3.9
1	D	148	ASP	3.9
1	D	123	SER	3.8
1	B	102	ASN	3.7
2	C	11	DA	3.7
1	B	43	ALA	3.6
2	C	10	DG	3.6
1	B	148	ASP	3.6
1	B	201	SER	3.2
1	B	208	CYS	2.9

Continued on next page...

CONFIDENTIAL VALIDATION REPORT

Continued from previous page...

Mol	Chain	Res	Type	RSRZ
1	B	224	LYS	2.8
1	B	178	GLY	2.7
4	A	10	DC	2.6
1	B	106	ALA	2.5
1	B	198	GLY	2.5
1	B	271	MET	2.5
1	B	200	ALA	2.4
1	B	221	ARG	2.4
1	D	266	PHE	2.4
1	D	211	LEU	2.3
1	B	123	SER	2.3
1	D	199	LEU	2.2
1	B	93	CYS	2.2
1	D	200	ALA	2.2
1	B	94	LEU	2.2
1	B	211	LEU	2.1
1	D	282	LEU	2.1
1	B	234	PRO	2.0
1	B	112	PRO	2.0

6.2 Non-standard residues in protein, DNA, RNA chains (i)

There are no non-standard protein/DNA/RNA residues in this entry.

6.3 Carbohydrates (i)

There are no monosaccharides in this entry.

6.4 Ligands (i)

In the following table, the Atoms column lists the number of modelled atoms in the group and the number defined in the chemical component dictionary. The B-factors column lists the minimum, median, 95th percentile and maximum values of B factors of atoms in the group. The column labelled 'Q< 0.9' lists the number of atoms with occupancy less than 0.9.

Mol	Type	Chain	Res	Atoms	RSCC	RSR	B-factors(Å^2)	Q<0.9
5	MN	D	401[A]	1/1	1.00	0.06	24,24,24,24	1

CONFIDENTIAL VALIDATION REPORT

6.5 Other polymers (i)

There are no such residues in this entry.

CONFIDENTIAL VALIDATION REPORT
